# Bimetallic Mixed Clusters Highly Loaded on Porous 2D Graphdiyne for Hydrogen Energy Conversion

**DOI:** 10.1002/advs.202102777

**Published:** 2021-09-08

**Authors:** Yang Gao, Yurui Xue, Taifeng Liu, Yuxin Liu, Chao Zhang, Chengyu Xing, Feng He, Yuliang Li

**Affiliations:** ^1^ Key Laboratory of Organic Solids Institute of Chemistry Chinese Academy of Sciences Beijing 100190 P. R. China; ^2^ Science Center for Material Creation and Energy Conversion Institute of Frontier and Interdisciplinary Science School of Chemistry and Chemical Engineering Shandong University Jinan 250100 P.R. China; ^3^ The Education Ministry Key Lab of Resource Chemistry Joint International Research Laboratory of Resource Chemistry Ministry of Education, and Shanghai Key Laboratory of Rare Earth Functional Materials College of Chemistry and Materials Science Shanghai Normal University Shanghai 200234 China; ^4^ University of Chinese Academy of Sciences Beijing 100049 P. R. China

**Keywords:** electrocatalysis, graphdiyne, hydrogen energy conversion, regulation of metal valence, 2D carbon allotrope

## Abstract

There is no doubt that hydrogen energy can play significant role in promoting the development and progress of modern society. The utilization of hydrogen energy has developed rapidly, but it is far from the requirement of human. Therefore, it is very urgent to develop methodologies and technologies for efficient hydrogen production, especially high activity and durable electrocatalysts. Here a bimetallic oxide cluster on heterostructure of vanadium ruthenium oxides/graphdiyne (VRuO*_x_*/GDY) is reported. The unique acetylene‐rich structure of graphdiyne achieves outstanding characteristics of electrocatalyst: i) controlled preparation of catalysts for achieving multiple‐metal clusters; ii) regulation of catalyst composition and morphology for synthesizing high‐performance catalysts; iii) highly active and durable hydrogen evolution reaction (HER) properties. The optimal porous electrocatalyst (VRu_0.027_O*_x_*/GDY) can deliver 10 mA cm^−2^ at low overpotentials of 13 and 12 mV together with robust long‐term stability in alkaline and neutral media, respectively, which are much smaller than Pt/C. The results reveal that the synergism of different components can efficiently facilitate the electron/mass transport properties, reduce the energy barrier, and increase the active site number for high catalytic performances.

## Graphical abstract

A general method is established to controllably induce bimetallic cluster growth using graphdiyne special structure, realizing the controllable regulation of the catalyst composition and morphology of the bimetallitic mixed clusters. This bimetallic oxide cluster on heterostructures of vanadium‐ruthenium oxides/graphdiyne (VRuO*_x_*/GDY) shows highly active and durable hydrogen evolution reaction (HER) performances much better than that of commercial Pt/C.

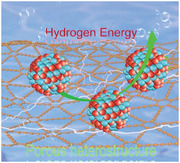

## 1 Introduction

Electrocatalytically splitting water into hydrogen (2H_2_O → 2H_2_ + O_2_) is one of the most promising strategies for large‐scale producing clear and potential fuel for renewable energy system.^[^ ^1^, ^2^, ^3^, ^4^, ^5^ ^]^ However, the unfavorable thermodynamic and sluggish kinetics of the water splitting process, especially under alkaline or neutral conditions, leads to the overall energy‐intensive process and requires high applied overpotentials. Platinum (Pt)‐based materials commonly exhibit the best hydrogen evolution reaction (HER) activity, however, the high cost and natural scarcity greatly limit their widespread applications. Recently, extensive efforts have been made to fabricate earth‐abundant elements based active and stable HER electrocatalysts based on, such as transition metal oxides (TMOs),^[^ ^6^ ^]^ phosphides,^[^ ^7^ ^]^ and sulfides,^[^ ^8^ ^]^ etc. However, the unfavorable hydrogen adsorption energy and poor intrinsic conductivity, making it difficult to achieve high electrocatalytic performance. The catalytic activity and stability of catalyst are strongly correlated with their electronic structures,^[^ ^9^ ^]^ electrical conductivity,^[^ ^10^ ^]^ interface effect,^[^ ^11^ ^]^ and active sites,^[^ ^12^ ^]^ etc. According to literatures, multi‐component metal catalysts with adjustable electronic structure and composites generally show higher catalytic performance than mono‐component ones.^[^ ^13^ ^]^ For example, Li and co‐workers reported that the bimetallic iron‐cobalt layered double‐hydroxide showed enhanced HER catalytic performances because of the greatly facilitated charge transport kinetics, increased electrochemical active surface area and number of active sites.^[^ ^14^ ^]^ Liu et al. reported cobalt–molybdenum carbide catalysts exhibited improved HER activity after the bimetallic alloying than monometallic ones.^[^ ^15^ ^]^ Recenlty, many Ruthenium (Ru) based catalysts have been reported and showed promising catalytic performances for HER under various conditions, such as Ru‐RuO*_x_*/CNT exhibited an ultrahigh activity toward HER in pH‐universal medium.^[^ ^16^ ^]^ The RuRh_2_ bimetallene nanoring showed an high activity in the both alkaline and neutral media, outperforming that of Pt catalysts and other reported HER catalysts.^[^ ^17^ ^]^ Another major challenge for developing efficient catalysts is their instability in aqueous electrolytes, especially when highly acidic or alkaline electrolytes are used. Therefore, neutral electrolyte is also an ideal alternative to petrochemical energy. The uniform growth of a catalyst on the conductive substrate is another effective route to boost the HER activity. The controllably loading of metal clusters on supports has become a research frontier in the current catalytic field, however, it still has many important scientific and bottleneck issues, which need to be solved urgently. A major challenge is how to control the loading of bimetallic mixed clusters, realize the improvement of the activity of catalysts and finally achieve transformative performance in the catalytic process.

Graphdiyne (GDY), a new 2D carbon allotrope containing *sp*‐ and *sp^2^*‐hybridized carbon atoms featuring uniformly triangular pores, uneven distribution surface charge, superior electrical conductivity and high stability, has been demonstrated as a promising electrode material applied in numerous fields from catalysis to energy conversion and storage.^[^ ^18^, ^19^, ^20^, ^21^, ^22^, ^23^, ^24^, ^25^, ^26^, ^27^, ^28^, ^29^, ^30^, ^31^, ^32^, ^33^, ^34^, ^35^, ^36^, ^37^, ^38^, ^39^, ^40^, ^41^, ^42^, ^43^, ^44^, ^45^ ^]^ The well‐defined porous structure of GDY endow it with numerous unique properties superior to traditional carbon materials. For example, the presence of sp‐C atoms (−C≡C−) in porous GDY leads to the formation of infinite active sites and highly intrinsic activity in catalysis. The natural cavity structure of porous GDY can facilitate the mass transport during the catalysis and simultaneously increase the catalytically active surface area. Our experimental and theoretical results have also demonstrated the GDY can act as an ideal support for the rationally fabrication of ideal interface structure toward highly active and stable catalysis, including GDY‐based zero‐valent atomic catalysts (Fe/GD,^[^ ^39^ ^]^ Ni/GD,^[^ ^39^ ^]^ Mo^0^/GDY^[^ ^40^ ^]^), heterostructured catalysts (eGDY/MoS_2_^[^ ^41^ ^]^ and e‐ICLDH@GDY/NF^[^ ^14^ ^]^), quantumn dots (OsO*_x_* QDs/GDY^[^ ^24^ ^]^), and metal‐free catalysts (p‐FGDY/CC^[^ ^29^ ^]^).

Herein, a series of porous 2D GDY loaded vanadium‐ruthenium oxides with different metal mole ratios have been synthesized, and used for HER. Remarkably, the as‐prepared catalysts have high catalytic activity in both basic and neutral conditions. When the Ru/V ratio is 0.027, the catalyst (VRu_0.027_O*_x_*/GDY) possessed the highest electrocatalytic activity with the smallest overpotentials of 13 and 12 mV at 10 mA cm^−2^ in alkaline and neutral conditions, respectively, and negligible activity loss after long‐term stability tests under respective conditions. Theoretical and experimental results reveal that such excellent catalytic activity is mainly attributed to the unique porous structure of the GDY‐based heterostructure and greatly enhanced charge transfer between GDY and clusters, which could facilitate the water dissociation process and result in the optimized the free energy for hydrogen generation.

## 2 Results and Discussion

**Figure 1:**
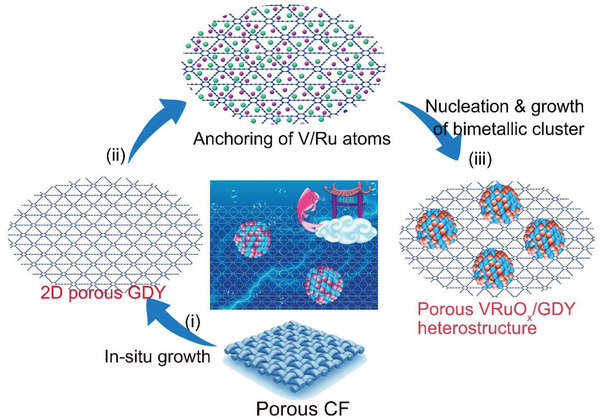
Schematic illustration of the synthesis route for VRuO*_x_*/GDY. Via a hydrothermal reaction, a series of GDY supported vanadium‐ruthenium oxides with different metal mole ratios have been synthesized, and used for HER.

**Figure 2:**
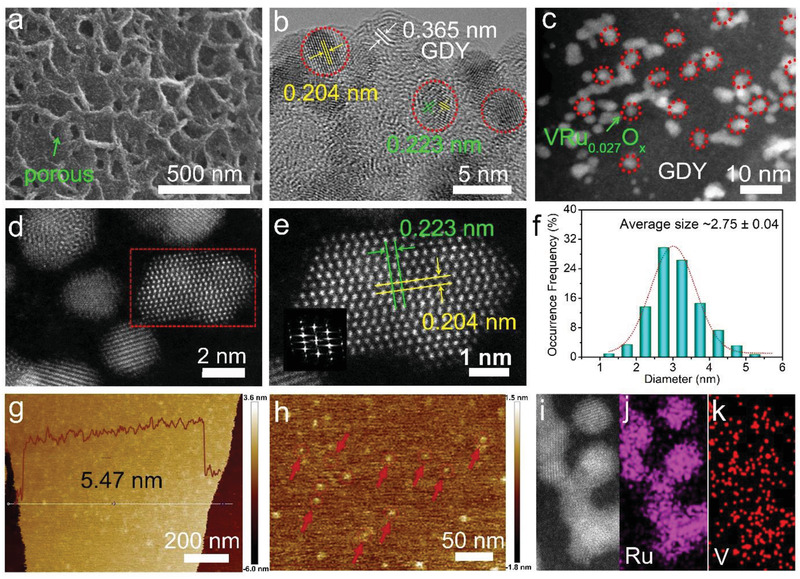
Structure characterization. a) SEM and b) HRTEM images of VRu_0.027_O*_x_*/GDY. c–e) HAADF‐STEM images of VRu_0.027_O*_x_*/GDY. f) Size distribution of VRu_0.027_O*_x_* clusters on porous GDY. g) Low‐ and h) high‐magnification of AFM images of VRu_0.027_O*_x_*/GDY. i) STEM image and corresponding elemental mapping images of j) Ru, and k) V.

**Figure** **1** schematically illustrates the synthetic routes to the rational synthesis of porous 2D GDY loaded bimetallic vanadium‐ruthenium oxide clusters (VRuO*_x_*/GDY, see details in the Experimental Section), including the first in‐situ growth of porous GDY electrodes (Figure S1a, Supporting Information) using the 3D porous carbon cloth fiber (CF) as the growing substrates (Figure S2, Supporting Information), followed by the adsorption of V^3+^ and Ru^3+^ ions on GDY surface. After the subsequent hydrothermal reaction, VRuO*_x_* clusters were grown on the surface of GDY (**Figure** **2**a–e). The high‐resolution transmission electron microscopy (HRTEM) images (Figure S1b–d, Supporting Information) show the interlayer distance of GDY was 0.365 nm. The scanning electron microscopy (SEM) and HRTEM images clearly showed the successful growth of VO*_x_*/GDY (Figure S1e–h, Supporting Information) and RuO*_x_*/GDY (Figure S1i–l, Supporting Information) on the surface of GDY. The average size of the VO*_x_* and RuO*_x_* clusters of VO*_x_*/GDY and RuO*_x_*/GDY was presented in Figure S3, Supporting Information. HRTEM demonstrated the crystalline nature of the VO*_x_*/GDY and RuO*_x_*/GDY with the interplanar spacing of 0.204 and 0.223 nm corresponding to the (002) plane of VO*_x_* (Figure S1h, Supporting Information) and (111) plane of RuO*_x_* species (Figure S1l, Supporting Information), respectively.

For VRu_0.027_O*_x_*/GDY, nano‐sized nanoparticles were successfully synthesized and uniformly distributed on GDY surface (Figure 2a,b, and Figure S4, Supporting Information). With the further increasing of the Ru/V ratio, aggregated particles grown on GDY surface were observed (Figure S5m–p, Supporting Information). The high‐angle annular dark field scanning TEM (HAADF‐STEM) images of VRu_0.027_O*_x_*/GDY in Figure 2c–e shows the uniform distribution of VRu_0.027_O*_x_* cluster on GDY. HRTEM (Figure 2b) and HAADF‐STEM (Figure 2e) images of VRu_0.027_O*_x_*/GDY reveal the presence of lattice fringes with interplanar spacings of 0.204 and 0.223 nm, corresponding to the (002) and (111) planes of VRu_0.027_O*_x_*. The average size of the VRu_0.027_O*_x_* clusters of VRu_0.027_O*_x_*/GDY was about 2.75 ± 0.04 nm, as indicated by the particle distribution in the Figure S3, Supporting Information and Figure 2f. Atomic force microscopy (AFM) results showed that the VRu_0.027_O*_x_*/GDY have the average thickness of 5.47 nm (Figure 2g). The formation VRu_0.027_O*_x_* clusters that marked by red arrows distributed heterogeneously on GDY surface without agglomeration (Figure 2h). EDS‐HAADF mapping results (Figures 2i–k) further confirmed the Ru and V were distributed in the form of VRu_0.027_O*_x_* clusters on GDY surface. The concentration of Ru of VRu_0.027_O*_x_*/GDY is about 2.33 at% (Figure S6, Supporting Information). The scanning TEM image (STEM) and EDX mapping images of VRu_0.027_O*_x_*/GDY (Figure S7, Supporting Information), VO*_x_*/GDY (Figure S8, Supporting Information) and RuO*_x_*/GDY (Figure S9, Supporting Information) also confirmed their elements distribution.

**Figure 3:**
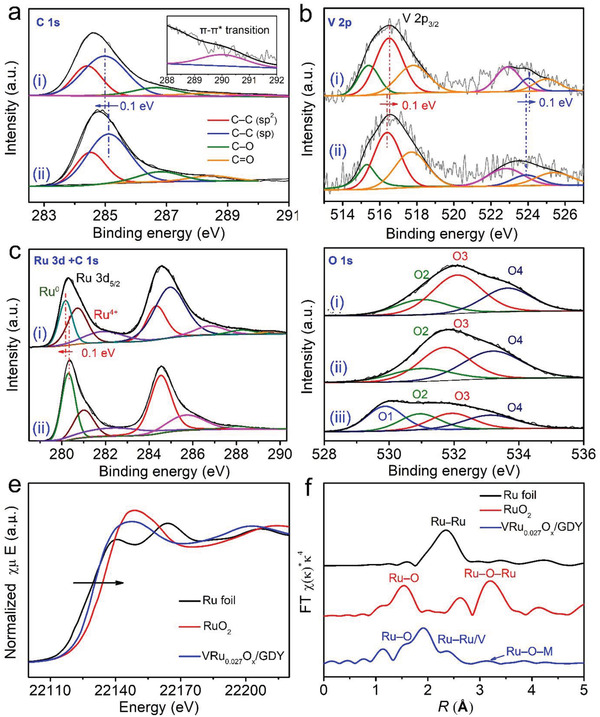
a) C 1s XPS spectra of i) VRu_0.027_O*_x_*/GDY and ii) GDY. b) V 2p spectra of i) VRu_0.027_O*_x_*/GDY and ii) VRu_0.027_O*_x_*. c) Ru 3d and C 1s spectra of i) VRu_0.027_O*_x_*/GDY and ii) VRu_0.027_O*_x_*. d) O 1s spectra of i) VO*_x_*/GDY, ii) RuO*_x_*/GDY, and iii) VRu_0.027_O*_x_*/GDY, respectively. e) The XANES spectra and f) Fourier‐transformed Ru K‐edge EXAFS spectra of Ru foil, RuO_2_, and VRu_0.027_O*_x_*/GDY.

X‐ray photoelectron spectroscopy (XPS) analysis was further performed to analyze the surface composition and chemical valences of samples (**Figure** **3**a–d and Figure S10, Supporting Information). As shown in Figure 3a, the C 1s XPS spectra of VRu_0.027_O*_x_*/GDY can be deconvoluted into five peaks at 284.4 (sp^2^‒C), 285.0 (sp‒C), 286.7 (C‒O), 288.4 (C═O), and 290.0 eV (*π*‒π* transition), respectively. The integration area of the sp‐ and sp^2^‐hybridized carbon is 2, in accordance with the GDY structure (Figure 3a, Table S1, Supporting Information). Besides, the C 1s XPS spectra of VRu_0.027_O*_x_*/GDY shows a negative shift by 0.10 eV, as compared to pure GDY, revealing the obvious charge transfer from VRu_0.027_O*_x_* complex to GDY. Figure 3b shows the V 2p XPS spectra of VRu_0.027_O*_x_*/GDY and VRu_0.027_O*_x_*. The V 2p XPS spectrum of VRu_0.027_O*_x_*/GDY could be divided into two spin‐orbital splitting and three shakeup satellites, corresponding to V^3+^ (515.4, 522.9), V^4+^ (516.5, 524.0), and V^5+^ (517.8, 555.0), respectively.^[^ ^46^ ^]^ The Ru 3d XPS spectrum of VRu_0.027_O*_x_*/GDY was associated with the overlap of orbits of the carbon elements. The peak located at 280.2 in the Ru 3d XPS spectra indicated the presence of Ru^0^ in VRu_0.027_O*_x_*.^[^ ^47^ ^]^ The peaks at 280.7 and 281.9 eV indicated the existence of Ru^4+^.^[^ ^42^ ^]^ The V 2p_3/2_ and Ru 3d_5/2_ XPS peaks for VRu_0.027_O*_x_*/GDY samples show the obvious positive and negative shifts in binding enerigies, as compared to pristine VRu_0.027_O*_x_* (Figures 3b,c), revealing the presence of obvious charge transfer in between V/Ru metal species and GDY.^[^ ^48^, ^49^ ^]^ Notably, such charge transfer during the different metals can result in the asymmetric distribution of electron, leading to the charge polarization of bimetallic component that is important for enhancing the HER activity.^[^ ^50^, ^51^ ^]^

The V 2p XPS spectra of catalysts with different V/Ru molar ratio and corresponding percentage of V^3+^, V^4+^, and V^5+^ were shown in Figures S11 and S12, Supporting Information. With the increasing of Ru to V ratios, the V2p_3/2_ peak exhibited a slightly positive shift from 516.4 eV (VRu_0.012_O*_x_*/GDY) to 516.5 eV (VRu_0.027_O*_x_*/GDY). The percentage of V^5+^ increased from 13.2% (VRu_0.012_O*_x_*/GDY) to 24.1% (VRu_0.017_O*_x_*/GDY) to 25.3% (VRu_0.022_O*_x_*/GDY) and to 28.8% for VRu_0.027_O*_x_*/GDY. With the further increasing of Ru/V ratio, the binding energy of V2p_3/2_ peak negatively shifted from 516.5 eV (VRu_0.027_O*_x_*/GDY) to 516.4 eV (VRu_0.042_O*_x_*/GDY), and the percentage of V^5+^ decreased to 24.3% (VRu_0.032_O*_x_*/GDY) to 23.4% (VRu_0.037_O*_x_*/GDY) to 21.6% (VRu_0.042_O*_x_*/GDY), respectively. These results showed that the mixed oxidation states of V (V^3+^, V^4+^, and V^5+^) plays the importance role in improving the HER catalytic performance. And the high‐valence V^5+^ could further modulate catalytic activity of the catalysts. These properties of VRu_0.027_O*_x_*/GDY are reported to improve the electrocatalytic activity.^[^ ^53^, ^54^ ^]^ For VO*_x_*/GDY, the V 2p_3/2_ in the V 2p spectrum and C‒O peaks in the C 1s spectrum exhibited slightly positive and negative shift, respectively, as compared to pristine VO*_x_* (Figure S13, Supporting Information) and GDY (Figure S14, Supporting Information). Similar electron transfer behaviors was also found in Ru 3d spectrum between RuO*_x_*/GDY and RuO*_x_*. The peaks at 281.4 and 282.6 eV of RuO*_x_*/GDY show a positive shift by 0.1 eV, as compared to RuO*_x_*, revealing the charge transfer from RuO*_x_* complex to GDY. (Figure S15, Supporting Information). The O 1s spectrum (Figure 3d and Figure S16, Supporting Information) of VO*_x_*/GDY, RuO*_x_*/GDY and VRu_0.027_O*_x_*/GDY showed three characteristic peaks at 531.0, 531.7‒532.1, and 533.1‒533.7 eV corresponding to oxygen atoms bound to metals (O2), surface‐adsorbed oxygen (O3), and adsorbed molecular water (O4), respectively.^[^ ^47^ ^]^ In addition to these peaks, the new peak at 529.9 eV in the O 1s spectrum of VRu_0.027_O*_x_*/GDY could be assigned to lattice oxygen of VRu_0.027_O*_x_*/GDY, which has been demonstrated to greatly enhance the catalytic activity.^[^ ^48^ ^]^ The percentage of lattice oxygen (O1), metal‐oxygen (O2), surface‐adsorbed oxygen (O3), and adsorbed molecular water (O4) based on the XPS spectrum of catalysts VO*_x_*/GDY, RuO*_x_*/GDY, and VRu_0.027_O*_x_*/GDY have been detailed calculated and presented in Table S2, Supporting Information.

To further investigate the electronic and coordination structures of VRu_0.027_O*_x_*/GDY, we conducted the X‐ray absorption near‐edge spectroscopy (XANES) and extended X‐ray absorption fine structure (EXAFS) measurements. Figure 3e and Figure S17, Supporting Information show the Ru and V K‐edge XANES profiles of the samples. Ru foil, RuO_2_, V foil, and VO_2_ were used as references. The absorption edge for VRu_0.027_O*_x_*/GDY is found to shift to a higher energy than that for Ru foil (Figure 3e) and V foil (Figure S17, Supporting Information), but all still smaller than the binding energies of RuO_2_ (Figure 3e) and VO_2_ (Figure S17, Supporting Information), respectively. EXAFS spectra (Figure 3f) for VRu_0.027_O*_x_*/GDY exhibited three prominent at around 1.55, 2.0, and 2.35 Å, corresponding to Ru–O bond, Ru‒C bond, and Ru–Ru/V bond, respectively.^[^ ^53^, ^54^ ^]^ In addition, some weak peaks at around 3.13 Å is associated with Ru–O–M (M = Ru or V) bond, which might be due to the existence of V–Ru mixed oxides. Raman spectroscopy was used to get more detailed information on the VRu_0.027_O*_x_*/GDY (Figure S18, Supporting Information). The presence of the peaks at 1956.9 and 2165.6 cm^−1^, which origianted from the vibration of the triple bonds for VRu_0.027_O*_x_*/GDY, demonstrated the integrity of the GDY in VRu_0.027_O*_x_*/GDY. Besides, the increasing of the intensity ratio of D and G bands (*I*_D_/*I*_G_) from 0.79 (GDY) to 0.88 (VRu_0.027_O*_x_*/GDY) suggested the formation of some new defect sites after the incorporation of GDY with VRu_0.027_O*_x_*. The X‐ray diffraction (XRD) patterns of GDY, VO*_x_*/GDY, RuO*_x_*/GDY, and VRu_0.027_O*_x_*/GDY (Figure S19, Supporting Information) show one strong and broad peak at about 27.0^o^ and 44.0^o^ ascribed to the diffraction from GDY. Very weak diffraction peak appeared in VO*_x_*/GDY, RuO*_x_*/GDY, and VRuO*_x_*/GDY XRD spectrum, indicating the formation of nanometer‐sized clusters on GDY surface.

**Figure 4:**
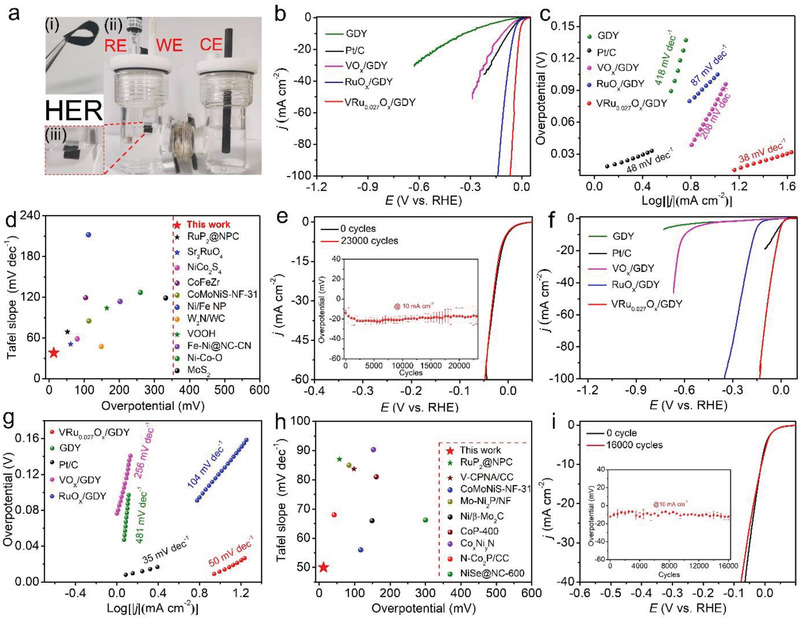
a) Photograph of the three‐electrode system (WE: working electrode; RE: reference electrode; CE: counter electrode) and iii) enlarged image of the WE in (ii). b) Polarization curves and c) corresponding Tafel slopes of the samples for HER in 1.0 m KOH. d) Comparison of the HER performances of the VRu_0.027_O*_x_*/GDY with reported electrocatalysts. e) Polarization curves of VRu_0.027_O*_x_*/GDY before and after 23 000 CV cycling tests in 1.0 m KOH (inset: cyclic voltammetry (CV) measurements of VRu_0.027_O*_x_*/GDY in 1.0 m KOH). f) Polarization curves and g) corresponding Tafel plots of the catalysts for HER in 1.0 m PBS. h) Comparison of the HER performances of the VRu_0.027_O*_x_*/GDY with the reported electrocatalysts in 1.0 m PBS. i) Polarization curves of VRu_0.027_O*_x_*/GDY before and after 16 000 CV cycling tests in 1.0 m PBS (inset: cyclic voltammetry (CV) measurements of VRu_0.027_O*_x_*/GDY in 1.0 m PBS).

The HER electrocatalytic performances of the catalysts were tested in H_2_‐saturated 1.0 m KOH using a standard three‐electrode system (**Figure** **4**a). As shown in Figure 4b,c, VO*_x_*/GDY and RuO*_x_*/GDY show relatively large overpotentials of 112 and 46 mV at 10 mA cm^−2^ (*η*_10_) and Tafel slopes of 208 and 87 mV dec^−1^, respectively. As the copresence of V and Ru species, the catalyst VRu_0.027_O*_x_*/GDY showed a greatly enhanceed catalytic activity (Figure 4b,c and Figure S20, Supporting Information). When the V/Ru mole ratio is 1:0.027, the obtained VRu_0.027_O*_x_*/GDY exhibited the best HER activity with the *η*_10_ of 13 mV and Tafel slope of 38 mV dec^−1^, respectively, which are much smaller than pure VRu_0.027_O*_x_* (*η*_10_ = 26 mV; Tafel slope = 73 mV dec^−1^), GDY (*η*_10_ = 340 mV; Tafel slope = 418 mV dec^−1^), commercial 20 wt% Pt/C (*η*_10_ = 79 mV; Tafel slope = 48 mV dec^−1^), and others VRuO*_x_*/GDY (Table S3, Supporting Information). Besides, it needs only an overpotential of 75 mV to reach a large current density of 100 mA cm^−2^, implying its promising nature for real industrial applications.^[^ ^55^, ^56^ ^]^ These values are much better than the recently reported electrocatalysts, for example, Sr_2_RuO_4_ (61 mV, 51 mV dec^−1^),^[^ ^49^ ^]^ NiRu‐LDH (166 mV, 107 mV dec^−1^),^[^ ^57^ ^]^ and NiCo_2_S_4_ (80 mV, 58.5 mV dec^−1^)^[^ ^58^ ^]^ (Figure 4d and Table S4, Supporting Information). The Tafel slope of 38 mV dec^−1^ for VRu_0.027_O*_x_*/GDY (Figure 4c) suggests that electrocatalyst proceeds a Volmer−Heyrovsky mechanism for HER in alkaline condition. Generally, V sites as 3d‐transion metal is benefical for cleaving the H—OH bond, but it suffers from poor ability of converting the resulting H_ad_* intermediates to H_2_.^[^ ^31^, ^32^ ^]^ Ru sites, with optimal M–H_ad_ energetics, could improve their HER kinetics by enhancing dissociative adsorption of water in the critical Volmer step.^[^ ^33^ ^]^ Therefore, VRu_0.027_O*_x_*/GDY, as a model catalyst, possesses favorable balance between facilitating water dissociation, V and Ru in close contact in VRu_0.027_O*_x_*/GDY promote the formation of H_ad_* intermediates on Ru sites through facilitated water dissociation.

The electrochemical stability is another important metric to assess the electrocatalyst performance. The stability of VRu_0.027_O*_x_*/GDY electrode was estimated by long‐term cyclic voltammetry (CV, Figure 4e) and chronoamperometry measurements (Figure S22, Supporting Information). Remarkably, the polarization curves of VRu_0.027_O*_x_*/GDY show a high current retention with a negligible current change after 23 000 CV scans (Figure 4e), and there is small decrease in the current density from 10 to 5.6 mA cm^−2^ after 18 h i‐t stability test at the potential of −0.022 V (vs RHE; Figure S22, Supporting Information). Further, SEM (Figures S23, Supporting Information), HRTEM (Figure S24, Supporting Information) and XPS (Figure S25, Supporting Information) analysis showed that the morphology, structure, and valence states for the composition of the catalyst have been preserved after the continuous CV test. These results indicated the long‐term stability of VRu_0.027_O*_x_*/GDY for HER in alkaline conditions.

We next investigated the electrocatalytic HER performance of VRu_0.027_O*_x_*/GDY in H_2_‐saturated 1.0 m PBS solution. As shown in Figure 4f,g, Figure S26a, Supporting Information, Table S5, Supporting Information, VRu_0.027_O*_x_*/GDY shows the best catalytic activity with the smallest overpotentials of 12 and 80 mV at 10 and 50 mA cm^−2^, respectively, and the smallest Tafel slope of 50 mV dec^−1^ among VRu_0.027_O*_x_* (58 mV dec^−1^), RuO*_x_*/GDY (145 mV dec^−1^), RuO*_x_* (104 mV dec^−1^), VO*_x_*/GDY (256 mV dec^−1^), VO*_x_* (423 mV dec^−1^), GDY (481 mV dec^−1^), and CC (552 mV dec^−1^) (Figures 4g, and Figure S26b, Supporting Information). These values are also much better than all prepared electrocatalysts (Figures S27, S28, Supporting Information) and almost all reported HER electrocatalysts in neutral conditions (Figure 4g and Table S6, Supporting Information). The long‐term stability of VRu_0.027_O*_x_*/GDY were further tested. It was observed that only a negligible change in current density occurred after 16 000 continuous CV cycles (Figure 4i). The superior HER activity of VRu_0.027_O*_x_*/GDY in neutral conditions could be attributed to the optimized electronic structure and enriched active sites. The stability of VRu_0.027_O*_x_*/GDY for HER was also tested and showed a decrease in current density from 10 to 2 mA cm^−2^ at the overpotential of 13 mV at 10 mA cm^−2^ after 18 h i‐t stability test. (Figure S29, Supporting Information). SEM images (Figure S30, Supporting Information), TEM (Figure S31, Supporting Information) and XPS (Figure S32, Supporting Information) results of VRu_0.027_O*_x_*/GDY demonstrated its high stability during HER process in 1.0 m PBS.

**Figure 5:**
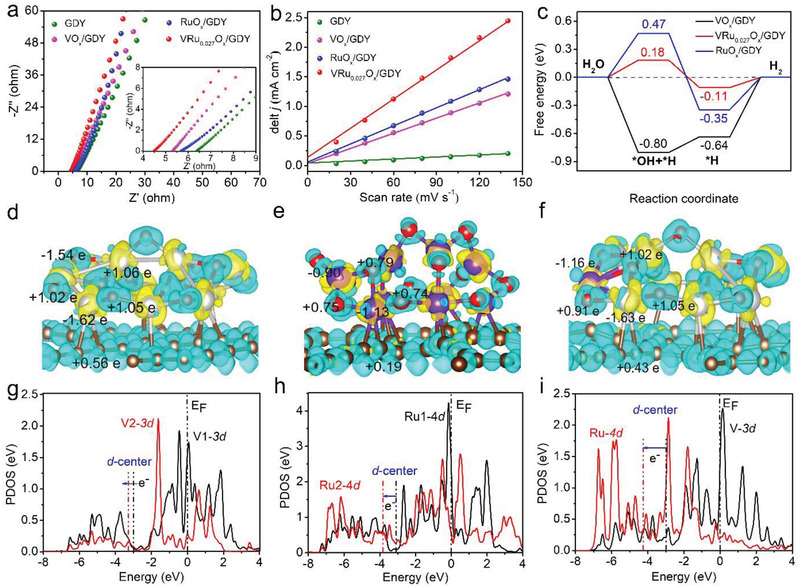
a) Nyquist plots of the catalysts. b) Current density differences against scan rates. c) Free energy diagram for HER on VO*_x_*/GDY, RuO*_x_*/GDY and VRu_0.027_O*_x_*/GDY (the insets represent the intermediates of adsorbed *OH+*H and *H on VRuO*_x_*/GDY catalyst surface). Charge distribution in d) VO*_x_*/GDY, e) and f) VRu_0.027_O*_x_*/GDY (Brown, silver, purple, and red balls represent C, V, Ru, and O atoms, respectively). Projected density of state (PDOS) of g) VO*_x_*/GDY, h) RuO*_x_*/GDY and i) VRu_0.027_O*_x_*/GDY (the positions of Fermi level (*E*_F_) and d band center of metal atoms are marked with dotted lines).

Electrical impedance spectra (EIS) analysis, electrochemical active surface area (ECSA) and density functional theory (DFT) calculations were carried out to determine the origin of catalytic activity of the as‐prepared samples. Nyquist plots were fitted to a R(QR) (QR) equivalent‐circuit model containing resolution resistance (*R*_s_) and charge transfer resistance (*R*_ct_). According the fitting results (**Figure** **5**b, Figure S33b, Supporting Information and Table S8, Supporting Information), the VRu_0.027_O*_x_*/GDY has the smallest *R*_s_ (4.95 Ω) and *R*_ct_ (50.3 Ω) among the catalysts, confirming the highest conductivity and the most facilitated proton and electron transfer process in water splitting. The ECSA of the catalysts was estimated by determining the double‐layer capacitance (*C*_dl_) via cyclic voltammetry (CV) method (Figurse S34, S36a–c, and S37a–c, Supporting Information). By scanning the cycle voltammetry (CV) curves between 0.02 and 0.12 V for varying sweep speeds from 20 to 140 mV s^−1^, a linear fitting was obtained between the sweep speed and current density. VRu_0.027_O*_x_*/GDY exhibited the lagest *C*_dl_ value of 17.2 mF cm^−2^ (Figure 5b and Figure S35, Supporting Information) among all prepared samples. For calculating the ESCA, we use specific capacitances (*C*_s_) of 0.04 mF cm^−2^. The ESCA value for VRu_0.027_O*_x_*/GDY was 430 cm^2^, which is larger than that of the pristine RuO*_x_*/GDY (266 cm^2^), RuO*_x_* (255 cm^2^), VO*_x_*/GDY (210 cm^2^), VO*_x_* (129 cm^2^), VRu_0.027_O*_x_* (319 cm^2^), GDY (35 cm^2^) and CF (8 cm^2^), respectively. These results confirmed the largest amounts of the active sites for VRu_0.027_O*_x_*/GDY, benefiting to the electrocatalytic activity. Furthermore, the *C*_dl_ values of VRu_0.012_O*_x_*/GDY, VRu_0.017_O*_x_*/GDY, VRu_0.022_O*_x_*/GDY, VRu_0.032_O*_x_*/GDY, VRu_0.037_O*_x_*/GDY, and VRu_0.042_O*_x_*/GDY were found to be 11.3, 12.9, 15.3, 13.7, 12.0, and 10.8 mF cm^−2^, respectively, which is smaller than VRu_0.027_O*_x_*/GDY (17.2 mF cm^−2^). The specific activity of the catalysts was then calculated based on the ECSA (Table S9, Supporting Information). For example, at *η* = −50 mV for HER, the intrinsic activities of the VRu_0.012_O*_x_*/GDY, VRu_0.017_O*_x_*/GDY, VRu_0.022_O*_x_*/GDY, VRu_0.027_O*_x_*/GDY, VRu_0.032_O*_x_*/GDY, VRu_0.037_O*_x_*/GDY and VRu_0.042_O*_x_*/GDY are 0.0046, 0.0241, 0.0316, 0.0321, 0.0319, 0.0172, and 0.0123 mA cm^−2^, respectively. This phenomenon clearly established the deposition of a sparse or accumulated of nanoparticles on GDY surface resulted in the loss of active area. Mass activity is another critical criterion to evaluate the catalytic performance of a catalyst in practical uses.^[^ ^30^ ^]^ Normalized by mass loading (Figure S38, Supporting Information), VRu_0.027_O*_x_*/GDY exhibited higher mass activities toward HER than VRu_0.012_O*_x_*/GDY and VRu_0.042_O*_x_*/GDY in both alkaline and neutral conditions. DFT calculations were performed to investigate the electrocatalytic mechanism of VO*_x_*/GDY, RuO*_x_*/GDY, and VRu_0.027_O*_x_*/GDY towards HER. As shown in the free energy diagram (Figure 5c), the adsorption free energies of intermediates of hydrogen evolution on VO*_x_*/GDY are too negative, limiting the final desorption of hydrogen production. However, the adsorption of intermediates of water splitting on RuO*_x_*/GDY are too positive, disfavoring the formation of (OH+H)*. By contrast, both the corresponding free energies for water splitting and hydrogen evolution on the VRu_0.027_O*_x_*/GDY catalyst were much closer to the thermoneutral state (i.e., Δ*G* = 0 eV), thereby exhibit the highest electrocatalytic activities towards alkaline HER. HER in an alkaline electrolyte is considered to proceed via two steps, that is, electron‐coupled water molecule dissociates into H* and OH^−^ on the surface of a catalyst (Volmer step); the concomitant transformation of H* into molecular H_2_ (the Heyrovsky step: H* + H_2_O + e^−^→H* +OH^−^; Tafel step: H* + H*→H_2_, where * is the active site).^[^ ^60^, ^61^, ^62^, ^63^ ^]^ VRu_0.027_O*_x_*/GDY and RuO*_x_*/GDY have Tafel slopes of 38 and 87 mV dec^−1^ (Figure 4c), respectively. A Tafel slope for the Volmer, Heyrovsky, and Tafel step, as the rate‐determining step for HER, is expected to be ≈120, ≈40, and ≈30mV dec^−1^, respectively. Accordingly, the hydrogen evolution for VRu_0.027_O*_x_*/GDY and RuO*_x_*/GDY proceeded through the Volmer–Heyrovsky mechanism in alkaline electrolyte, and the Heyrovsky step as the rate‐determining step. Differential charge density distribution exhibited an obvious charge transfer from VO*_x_* (1.87 e, Figure 5d), RuO*_x_* (0.61 e, Figure 5e), and VRu_0.027_O*_x_* (1.84 e, Figure 5f) to GDY, respectively. The results were in accordance with the XPS analysis (Figure 3b,c and Figures S13–S15, Supporting Information). We further examined the charge distribution in VO*_x_*/GDY, RuO*_x_*/GDY and VRu_0.027_O*_x_*/GDY based on Bader charge analysis, as shown in Figure S39, Supporting Information. After being incorporated with GDY, the electrons in VO*_x_*, RuO*_x_*, and VRu_0.027_O*_x_* were redistributed, resulting in various valence states of metal atoms. An obvious electron loss on metal atoms (such as V and Ru) and electron gain on nonmetal atoms (such as O and C) were observed. It is also noted that the electron loss/gain on the metal/nonmetal sites in VRu_0.027_O*_x_*/GDY are more moderate compared to those in VO*_x_*/GDY and RuO*_x_*/GDY. The interesting charge‐transport behavior guarantee the high intrinsic activity of the catalyst. The projected density of state (PDOS, Figures 5g–i) shows that the gap of *d* band center position between adjacent V and Ru atoms is much larger than that between two adjacent V atoms or Ru atoms, showing the strong charge transfer and synergistic effect between V and Ru atoms, which played a significant role in facilitating the electrocatalytic performance of VRu_0.027_O*_x_*/GDY towards HER. The catalytic performances of these models for HER were well consistent with our experimental results.

## 3 Conclusion

In summary, the mechanism of the graphdiyne‐based bimetallic mixed clusters catalyzed HER has been elucidated. Our results reveal that graphdiyne can guide the formation of optimum interface structure with highly catalytic activity and durability for HER in both alkaline and neutral conditions. And the introduction of bimetal species could effectively tune the catalyst composition, electronic structure, and the number of the active sites, finally improving the intrinsic electrocatalytic activity. For example, the VRu_0.027_O*_x_*/GDY can reach 10 mA cm^−2^ at very small overpotentials of 13 and 12 mV in alkaline and neutral electrolyte, respectively, together with excellent long‐term stability. This study provides fundamental guidelines and new avenues for rational design and synthesis of highly active and robust catalysts with potential applications in the electrochemical water splitting devices.

## 4 Experimental Section

### Materials

Tetrabutylammonium fluoride (TBAF) was purchased from Alfa Aesar. Hexabromobenzene was brought from J&K Scientific. Toluene and tetrahydrofuran (THF) were refluxed with sodium pieces for sufficient time in order to remove the remaining water. All other reagents were purchased from Sinopharm Chemical Reagent Co., Ltd., and used without further purification unless specifically mentioned. The water used for all experiment was purified with a Millipore system. All the chemicals were of chemical grade and were used as received without further purification, weighed with MeTTLER TOLEDO electronic balance. Before synthesized GDY, the carbon cloth fiber (CF) need pretreated in boiled concentrated nitric acid, and supersonic in deionized water, acetone and deionized water, respectively. The copper foils could not only act as the catalyst but also provide a feasible platform for the direct growth of GDY with controlled structures. The copper foils could afford copper ions for the formation of copper‐pyridine complexes (catalyst) for catalyzing the acetylenic coupling reaction.^[^ ^21^ ^]^ Cu foils also need ultrasound in dilute hydrochloric acid, deionized water, and acetone, respectively.

### Synthesis of GDY

GDY was prepared by using previously reported method with minor modification.^[^ ^14^ ^]^ Several pieces of copper foil and carbon cloth fiber (CF, 2.0 cm × 3.0 cm) were added to pyridine solution (50 mL) at a three‐necked flask, which was heated at 110 °C for 2 h. 20 mg hexaethylbenzene (HEB) was dissolved in 50 mL pyridine and dropwise added to the three‐necked flask. And then heated at 110 °C for 12 h under Ar atmosphere. After the reaction, the obtained products were cleaned with hot acetone and DMF, the thoroughly cleaned with KOH (4 m), HCl (6 m), KOH (4 m) and deionized water, respectively.

### Synthesis of VO*_x_*/GDY, RuO*_x_*/GDY or VRuO*_x_*/GDY

VO*_x_*/GDY, RuO*_x_*/GDY or VRuO*_x_*/GDY were prepared through a simple hydrothermal reaction. First, 150 mg of VCl_3_ or 8 mg of RuCl_3_·*x*H_2_O (≥37%) was added to 15 mL deionized water, respectively, then stirred for 60 min. The resulted homogeneous solution was transferred into a 30 mL Teflon‐lined stainless‐steel autoclave. Subsequently, the GDY‐coated carbon cloth fiber (CF) was immersed in the solution. After being conducted by hydrothermal process at 150 °C for 7 h, the obtained VO*_x_*/GDY or RuO*_x_*/GDY was cleaned and immediately used for electrochemical tests. VRuO*_x_*/GDY was prepared according to the experimental steps described above. By modulating the molar ratios of RuCl_3_·*x*H_2_O (≥37%) and VCl_3_ (0.012: 1, 0.017: 1, 0.022: 1, 0.027:1, 0.032: 1, 0.037: 1, 0.042: 1), a series of VRuO*_x_*/GDY were achieved and named as VRu_0.012_O*_x_*/GDY, VRu_0.017_O*_x_*/GDY, VRu_0.022_O*_x_*/GDY, VRu_0.027_O*_x_*/GDY, VRu_0.032_O*_x_*/GDY, VRu_0.037_O*_x_*/GDY and VRu_0.042_O*_x_*/GDY, respectively. The catalysts were named directly by Ru/V molar ratios added during the synthesis process. And the exact of RhCl_3_ *x*H_2_O (≥37%) masses that were used in the preparation of VRuO*_x_*/GDY were 3.5 mg (VRu_0.012_O*_x_*/GDY), 5 mg (VRu_0.017_O*_x_*/GDY), 6.5 mg (VRu_0.022_O*_x_*/GDY), 8 mg (VRu_0.027_O*_x_*/GDY), 9.5 mg (VRu_0.032_O*_x_*/GDY), 11 mg (VRu_0.037_O*_x_*/GDY), 12.5 mg (VRu_0.042_O*_x_*/GDY), respectively.

### Synthesis of VO*_x_*, RuO*_x_*, or VRuO*_x_*

VO*_x_*, RuO*_x_*, and VRuO*_x_* was prepared according to the synthesis method of VO*_x_*/GDY, RuO*_x_*/GDY, and VRuO*_x_*/GDY, but with a minor modification, which need replace a GDY coated carbon cloth fiber with a bare carbon cloth.

### Characterization

Scanning electron microscopy (SEM) were recorded using an S‐4800 field emission scanning electron microscope. Transmission electron microscopy (TEM), high resolution transmission electron microscopy (HRTEM) images and elemental mapping results were obtained on a JEM‐2100F electron microscope operating at 200 kV. Atomic force microscope (AFM, Bruker Bioscope Catalyst) was used to characterize size and thickness of electrocatalysts. Raman spectra were measured through the Renishaw‐2000 Raman spectrometer exploiting a 473 nm excitation laser source. And a Thermo Scientific ESCALab 250Xi instrument with monochromatic Al K*α* X‐ray radiation was used to perform the X‐ray photoelectron spectroscope (XPS) measurement. X‐ray diffraction (XRD) was performed using a Japan Rigaku D/max‐2500 rotation anode X‐ray diffractometer and graphite‐monochromated Cu K*α* radiation (*λ* = 1.54178 Å). The content of Ru elements was measured by Inductive Coupled Plasma Mass Spectrometry (ICP‐MS) (Thermofischer).

### XANES and EXAFS Characterizations

XANES measurements were performed at the 1W1B beamline of the Beijing Synchrotron Radiation Facility. The XANES raw data were background‐subtracted, normalized, and Fourier‐transformed by standard procedures with the ATHENA program. Least‐squares curve fitting analysis of the EXAFS *χ*(k) data, including multiple shell contributions was carried out using the ARTEMIS program with the theoretical scattering amplitudes, phase shifts, and the photoelectron mean free path for all paths calculated by the ab initio code FEFF 6.0. The data were fitted in R‐space.

### Electrochemical Studies

For HER, all electrochemical experiments were conducted through an electrochemical workstation (CHI. 660E, Shanghai CH. Instruments, China) with a typical three‐electrode system. The as‐prepared catalysts were used as working electrode with geometric surface area (about 4 mm × 3 mm–4 mm × 5 mm). Graphite rod and saturated calomel electrode (SCE) were used as the counter electrode and reference electrode, respectively. Pt/C (20 wt%) was coated on a glassy carbon electrode (GCE, 0.07 cm^2^) as a reference sample for the HER. 1 mg of Pt/C (20 wt%) powder (Alfa Aesar) was first mixed with ethanol (950 uL) and 5 wt% Nafion solution (50 uL) under sonication for 2 h. The working electrode (Pt/C) was then prepared by drop casting 20 µL of the above solution onto the freshly cleaned GCE (mass loading: 0.286 mg cm^−1^). Before each electrochemical testing, electrolytes including 1.0 m KOH and 1.0 m PBS aqueous solutions were saturated by high‐pure H_2_ gas. The LSV polarization curves were proceeded in H_2_‐saturated electrolyte at 2 mV s^−1^ scanning rate. Cyclic voltammograms (CV) measurements were performed in an alkaline and neutral environment at 100 mV s^−1^ scanning rate in a potential ranges from −0.85 to −1.30 V and −0.4 to −0.85 V, respectively. The EIS data were gathered in the frequency range from 0.1 to 100 000 Hz at the fixed potential in the HER region (E < 0 V vs RHE) with a signal amplitude perturbation of 5 mV. The chronoamperometric tests were carried out at a constant overpotential to reach an initial current density of 10 mA cm^−2^. To study electrochemically active surface areas (ESCAs), CV measurements were performed in the non‐Faradaic region (0.05–0.12 V vs SCE) at different scan rates (20, 40, 60, 80, 100, 120, and 140 mV s^−1^) in 1.0 m KOH, and the derived double‐layer capacitance (*C*_dl_) was used to further assess the ESCA. The value of *C*_dl_ equals the slope of the fitting line of  *J*  =  (*J_a_* − *J_c_*)/2 against scan rates, while *J*_a_ and *J*_c_ represent the anodic and cathodic currents at 0.07 V versus SCE, respectively. We selected the 0.02–0.12 V (vs SCE) region to characterize the *C*_dl_ of the electrocatalyst because this is a non‐Faradaic region without redox reactions occurring, which makes the accurate evaluation of *C*_dl_ feasible.

The *C*_dl_ can be further converted into ESCA by usingequation:^[^ ^64^ ^]^

(1) $$\mathrm{ESCA}\;=\;R_{f}\times A_{\mathrm{geom}}$$

(2) $$R_{f}=C_{\mathrm{dl}}/C_{\mathrm{dl}},\mathrm{ideal}$$

where *C*_dl_,ideal is the double layer capacitance of an ideally flat electrode, which is usually taken as 40 µF cm^−2^ in alkaline media, and Ageom is the geometric surface are of the electrode.

The specific current densities (*j*_s_) for our catalysts can be calculated by dividing the current density per geometric area (*j*_g_) at a given overpotential by the determined roughness factor (*R*_f_), which was calculated by dividing electrochemically active surface area (ECSA) by geometric area of the electrode.

(3) $$j_{s}=\;\frac{j_{g}}{R_{f}}$$

The obtained LSV curves were corrected by the *iR*_s_ loss compensation according to the following Equation (4):^[^ ^65^ ^]^

(4) $$E_{\mathrm{corr}}=E_{\mathrm{mea}}\;-iR_{s}$$

where *E*_corr_ is the corrected potential, *E*_mea_ is the measured potential and *R*_s_ is the equivalent series resistance determined by electrochemical impedance spectroscopy (EIS). Unless otherwise specified, all potentials are converted to reversible hydrogen electrode (RHE) according to the Equation (5):^[^ ^65^ ^]^

(5) $$E\;\left(\mathrm{RHE}\right)=E_{\mathrm{mea}}\;-iR_{s}+0.059\times \mathrm{pH}+E^{0}\left(\mathrm{SCE}\right)$$

where *E* ^0^ (SCE) is 0.242 V.

### Computational Details

Density functional theory (DFT) calculations have been carried out using the Vienna ab‐initio simulation package (VASP). The Perdew‐Burke‐Ernzerbof (PBE) exchange and correlation functional is chosen.^[^ ^66^, ^67^, ^68^, ^69^ ^]^ The Blöchl's all‐electron‐like projector augmented wave (PAW) method is used to describe the interactions between valence electrons and ion cores.^[^ ^70^, ^71^ ^]^ The plane wave basis set kinetic cutoff energy of 450 eV with a Monkhost–Pack k‐point grid of 3 × 3 × 1 are applied.^[^ ^72^ ^]^ The electron occupancies were determined according to Fermi scheme with an energy smearing of 0.1 eV. The convergence tolerance of total energy calculation is determined at 1.0 × 10^−6^ eV atom^−1^ with ionic force minimization level of 0.01 eV Å^−1^. To avoid the periodic interactions, a vacuum layer as large as 20 Å is used along the c direction. The DFT‐D3 method was employed to consider the long‐range van der Waals (vdW) interactions.^[^ ^73^, ^74^ ^]^ The (4d, 5s, 5p), (3d, 4s, 4p), (2s, 2p), (1s) states are chosen as the valence states for Ru, V, C, O, and H atoms, respectively. DFT+U framework is imbedded within the VASP source code. The Hubbard U on the Ru‐4d and V‐3d are self‐consistently to be 3.06 and 3.40 eV, respectively.^[^ ^72^ ^]^ To meet the average particle size experimentally as close as possible, the cluster of VO*_x_* and VRu_0.027_O*_x_* (as example) with 23 atoms are employed to construct the model.^[^ ^75^ ^]^

The Gibbs free energies difference of intermediates involved in HER pathways are calculated by utilizing the computational hydrogen electrode model (6):

(6) ΔG=ΔE+ΔZPE−TΔS

where Δ*E* is the energy difference of adsorption. ΔZPE and *T*Δ*S* are the zero‐point energy correction term and the entropy correction term, respectively. The two terms are obtained by the frequency calculation at *T* = 300 K. The Gibbs free energy of (H^+^+e^−^) is equivalent to the energy of 1/2G_H2_ in the study.

ZPE values of adsorbed species is derived after the frequency calculation by Equation (7):^[^ ^75^ ^]^

(7) $$\mathrm{ZPE}\;=\frac{1}{2}\;\sum hv_{i}$$

*TS* values of adsorbed species is also calculated using the vibrational frequencies by Equation (8):

(8) $$TS_{v}=\;TK_{B}\left[\sum_{K}\ln\left(\frac{1}{1-e^{-hv_{K}/Tk_{B}}}\right)\right]+\sum_{K}\frac{hv_{K}}{Tk_{B}}\frac{1}{\left(e^{hv_{K}/Tk_{B}}-1\right)}$$

where *k*_B_ is the Boltzmann constant, *T* is Temperature, *K* is vibrational mode, *v* is vibrational frequency for the intermediates, which is obtained from DFT calculations.

## Conflict of Interest

The authors declare no conflict of interest.

## Supporting information

Supporting Information

## Data Availability

Research data are not shared.
