# Supplementary material for: Bimetallic Mixed Clusters Highly Loaded on Porous 2D Graphdiyne for Hydrogen Energy Conversion
Source: Adv Sci (Weinh). 2021 Sep 8;8(21):2102777. doi: 10.1002/advs.202102777 (PMC8564434; doi:10.1002/advs.202102777)
Supplement: Supplementary file 1 — Supporting Information [file ADVS-8-2102777-s001.pdf]

© 2021 Wiley-VCH GmbH

ADVANCED SCIENCE
Open Access

Supporting Information

for *Adv. Sci.*, DOI: 10.1002/advs.202102777

Bimetallic Mixed Clusters Highly Loaded on Porous 2D Graphdiyne for Hydrogen Energy Conversion

Yang Gao, Yurui Xue, Taifeng Liu,* Yuxin Liu, Chao Zhang, Chengyu Xing, Feng He* and Yuliang Li**

WILEY-VCH

Supporting Information

Bimetallic Mixed Clusters Highly Loaded on Porous 2D Graphdiyne for Hydrogen Energy Conversion

Yang Gao, Yurui Xue, Taifeng Liu,* Yuxin Liu, Chao Zhang, Chengyu Xing, Feng He* and Yuliang Li**

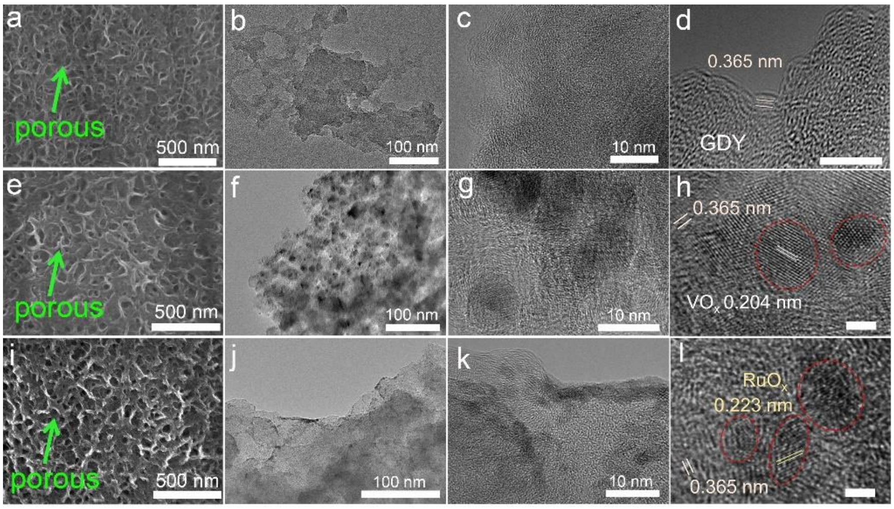

Figure S1. SEM, TEM and HRTEM images of a-d) GDY, e-h) VO_x/GDY, and i-l) RuO_x/GDY.

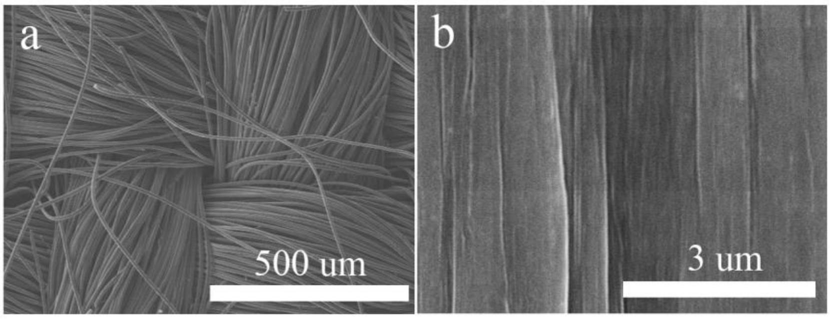

Figure S2. SEM image of the bare CF.

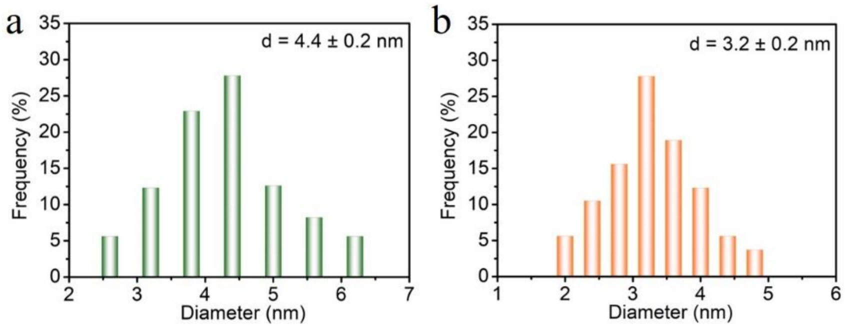

Figure S3. Size distribution histograms of a) VO_x and b) RuO_x clusters on GDY.

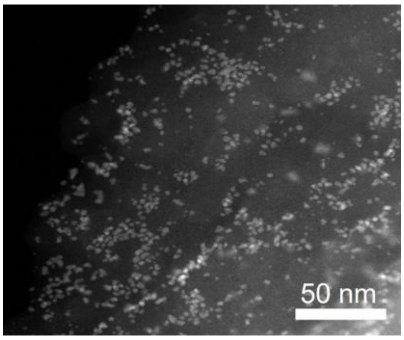

Figure S4. HAADT-STEM image of $VRu_{0.027}O_x$/GDY.

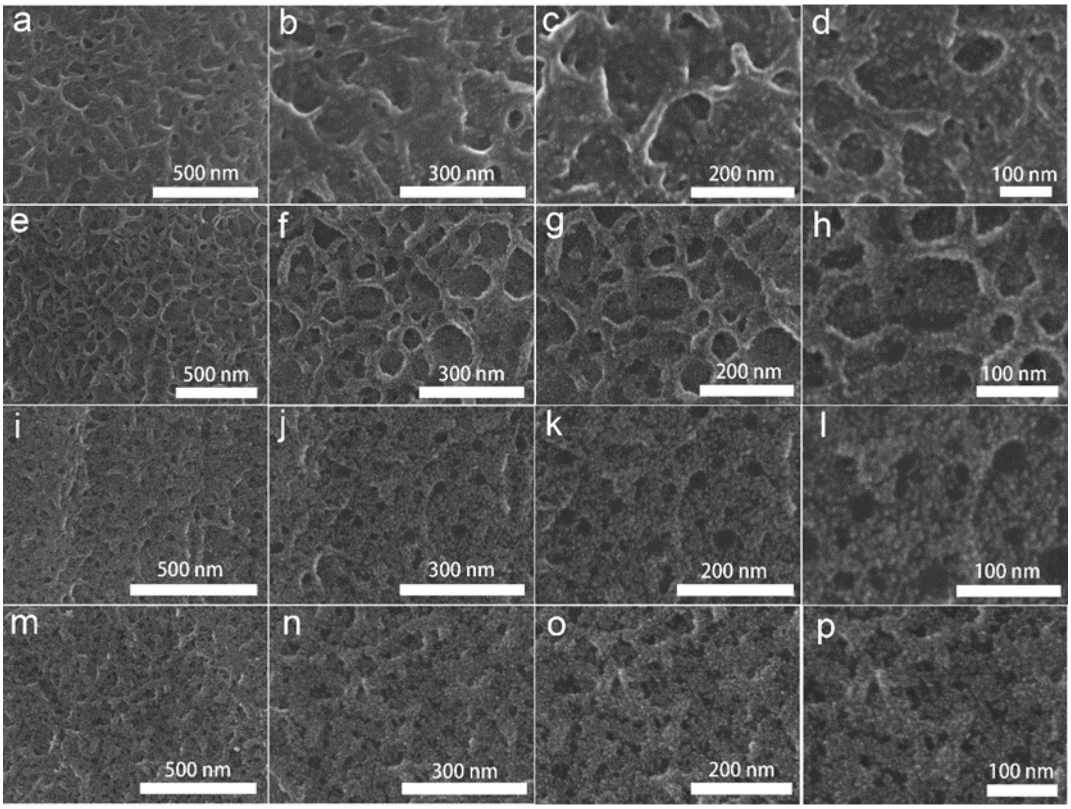

Figure S5. a-d) SEM images of $VRu_{0.012}O_x$/GDY. e-h) SEM images of $VRu_{0.022}O_x$/GDY. i-l) SEM images of $VRu_{0.032}O_x$/GDY. m-p) SEM images of $VRu_{0.042}O_x$/GDY.

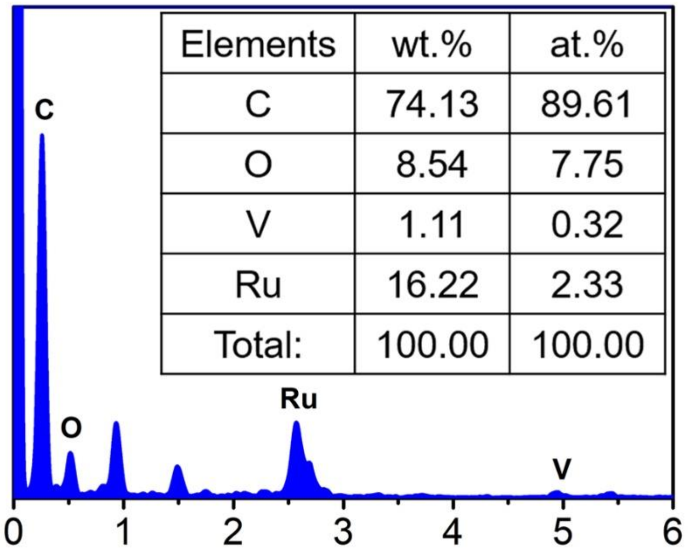

Elements	wt.%	at.%
C	74.13	89.61
O	8.54	7.75
V	1.11	0.32
Ru	16.22	2.33
Total:	100.00	100.00

Figure S6. EDS analysis of $VRu_{0.027}O_x$/GDY (Inset: the relative content of each element).

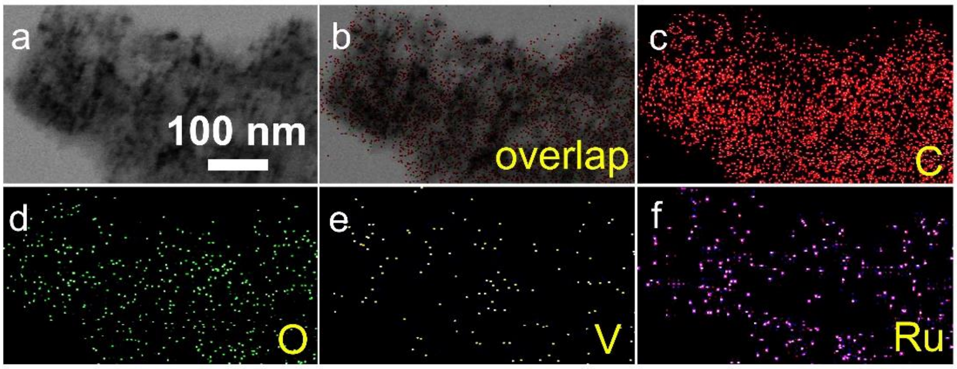

Figure S7. a) Scanning TEM measurements $VRu_{0.027}O_x$/GDY and corresponding elemental mapping images of c) C, d) O, e) V and f) Ru.

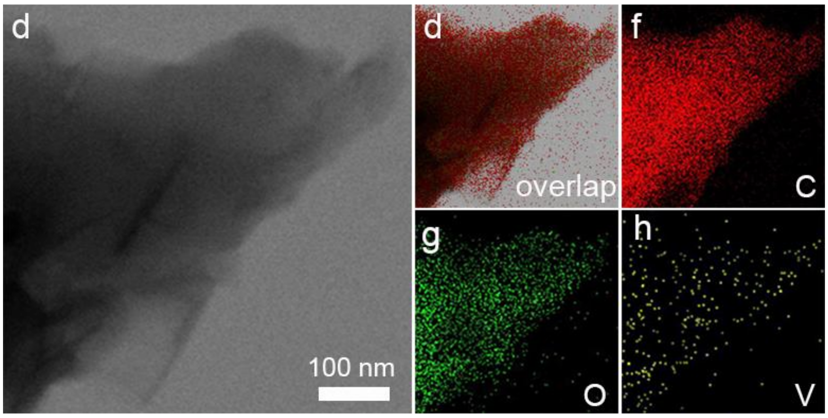

Figure S8. a) Scanning TEM measurements VO_x/GDY and corresponding elemental mapping images of c) C, d) O and e) V.

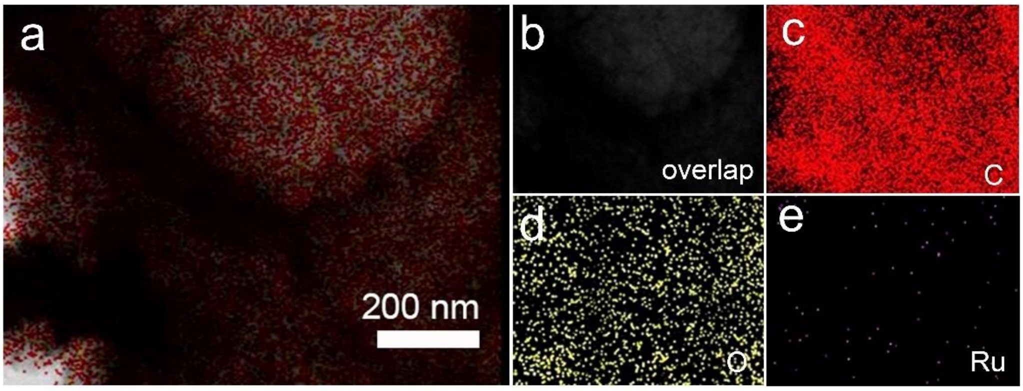

Figure S9. a) Scanning TEM measurements RuO_x/GDY and corresponding elemental mapping images of c) C, d) O and e) Ru.

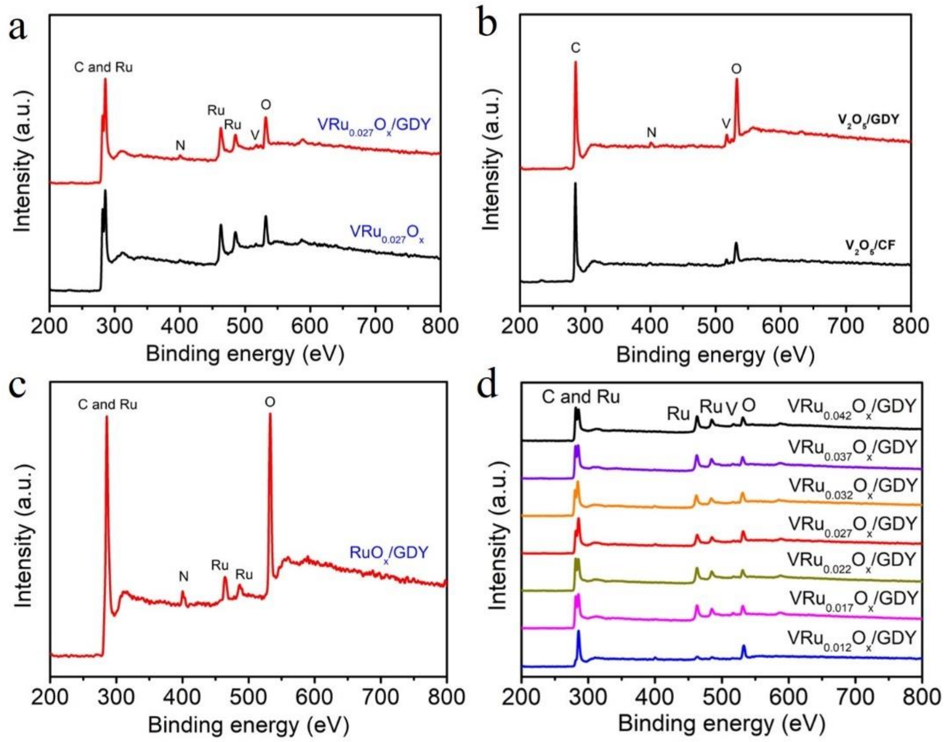

Figure S10. a) The XPS survey spectrum of $VRu_{0.027}O_x$/GDY and $VRu_{0.027}O_x$. b) The XPS survey spectrum of VO_x/GDY and VO_x. c) XPS survey of RuO_x/GDY. d) XPS survey spectrum of $VRuO_x$/GDY with different V/Ru ratio.

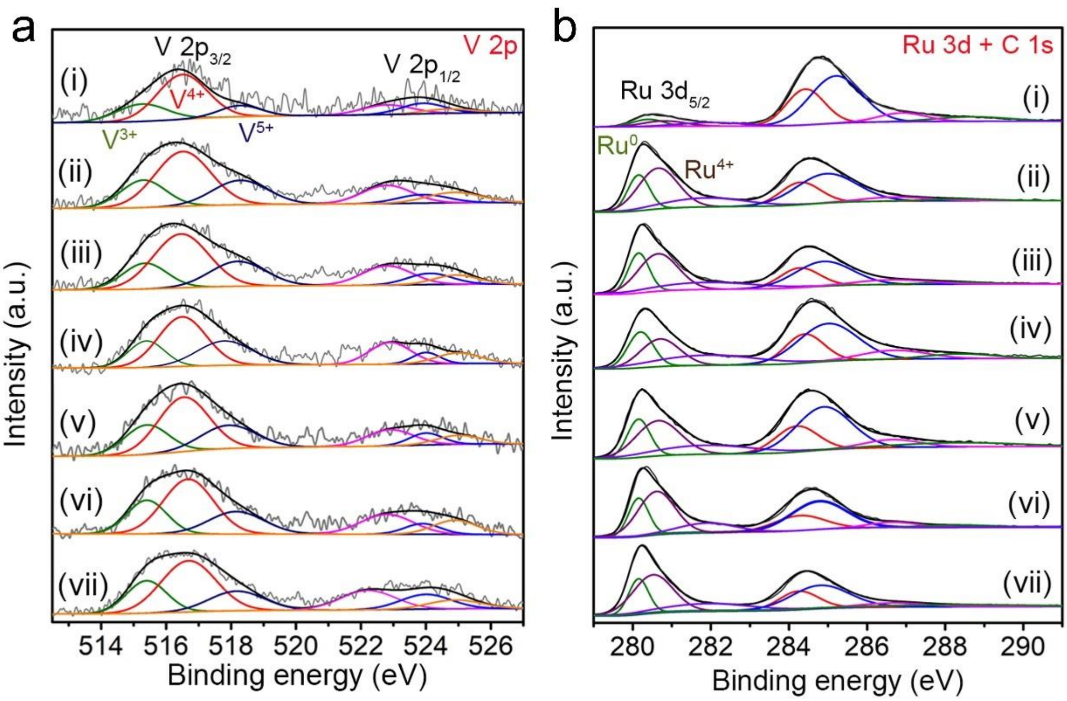

Figure S11. a) V2p and b) Ru 3d XPS spectra of (i) $VRu_{0.012}O_x$/GDY, (ii) $VRu_{0.017}O_x$/GDY, (iii) $VRu_{0.022}O_x$/GDY, (iv) $VRu_{0.027}O_x$/GDY, (v) $VRu_{0.032}O_x$/GDY, (vi) $VRu_{0.037}O_x$/GDY and (vii) $VRu_{0.042}O_x$/GDY.

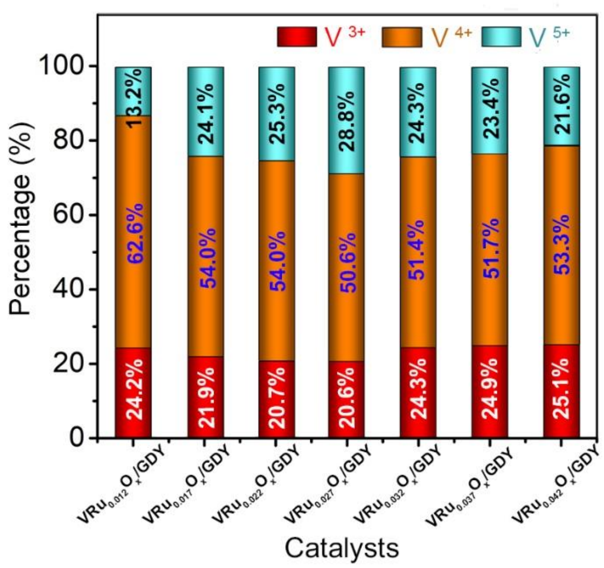

Figure S12. The percentage of V^{3+}, V^{4+} and V^{5+} of catalysts $VRu_{0.012}O_x$/GDY, $VRu_{0.017}O_x$/GDY, $VRu_{0.022}O_x$/GDY, $VRu_{0.027}O_x$/GDY, $VRu_{0.032}O_x$/GDY, $VRu_{0.037}O_x$/GDY and $VRu_{0.042}O_x$/GDY.

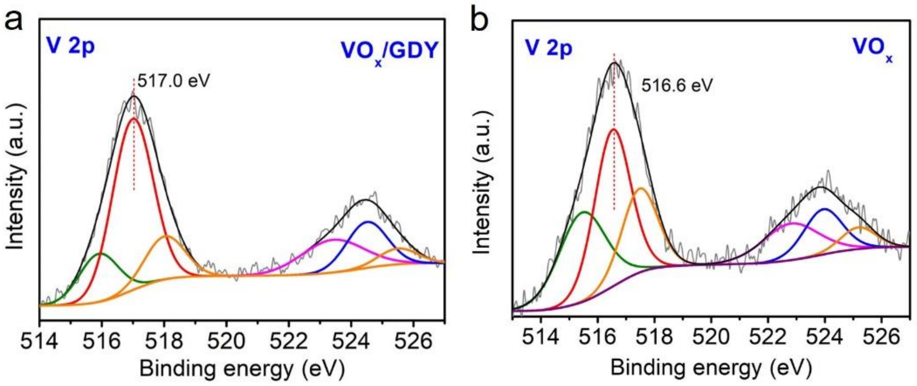

Figure S13. V 2p spectra of VO_x/GDY and VO_x.

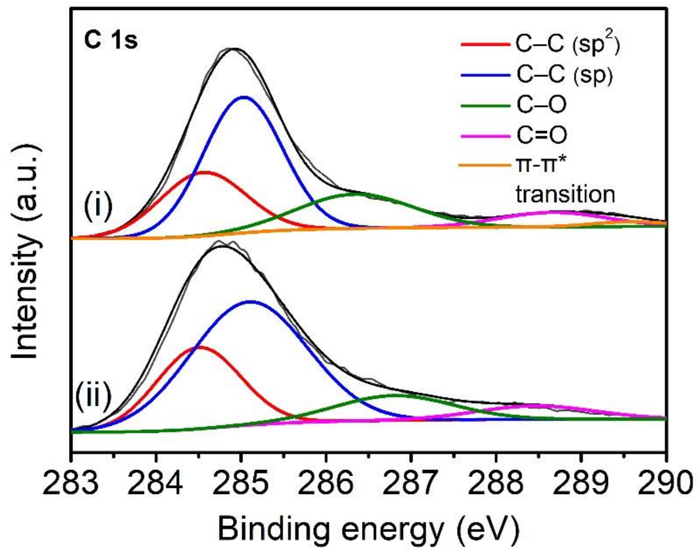

Figure S14. C 1s spectra of (i) VO_x/GDY and (ii) VO_x.

The C 1s XPS spectra of VO_x/GDY can be deconvoluted into four peaks at peaks at 284.5 (sp^2-C), 285.0 (sp-C), 286.4 (C-O), 288.7 eV (C=O) and 289.6 eV (π–π transition), respectively. The peak at 286.5 eV (C-O) of VO_x/GDY shows a negative shift by 0.40 eV as compared to pure GDY, revealing the obvious charge transfer from VO_x complex to GDY.

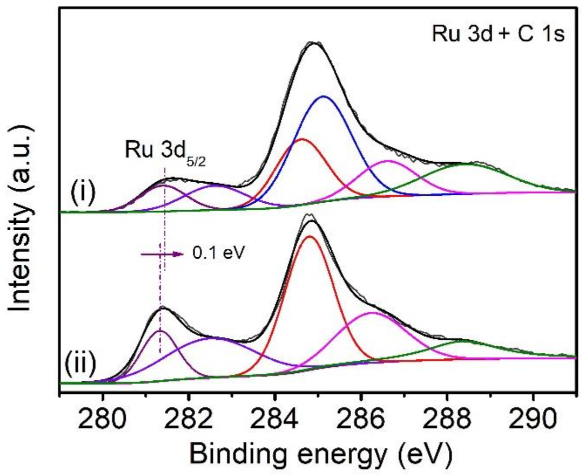

Figure S15. Ru 3d and C 1s XPS spectra of (i) RuO_x/GDY and (ii) RuO_x.

As shown in Figure S15, the peaks at 281.4 and 282.6 eV of RuO_x/GDY show a positive shift by 0.1 eV, as compared to RuO_x, revealing the charge transfer from RuO_x complex to GDY.

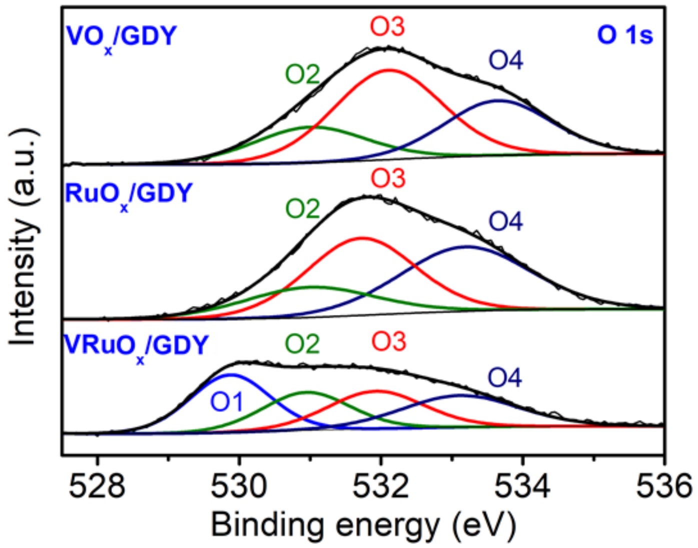

Figure S16. O 1s spectra of VO_x/GDY, RuO_x/GDY and $VRu_{0.027}O_x$/GDY.

For VO_x/GDY, the peaks at 531.0 (O2) , 532.1 (O3) and 533.7 eV (O4) correspond to oxygen atoms bound to metals, surface-adsorbed oxygen and adsorbed molecular water, respectively. For RuO_x/GDY, the peaks at 531.0 (O2), 531.7 (O3) and 533.2 eV (O4) correspond to oxygen atoms bound to metals, surface-adsorbed oxygen and adsorbed molecular water, respectively. For $VRu_{0.027}O_x$/GDY, the peaks located at 529.9 (O1), 531.0 (O2) , 531.9 (O3) and 533.1 eV (O4) correspond to lattice oxygen of $VRu_{0.027}O_x$/GDY, oxygen atoms bound to metals , surface-adsorbed oxygen and adsorbed molecular water, respectively.

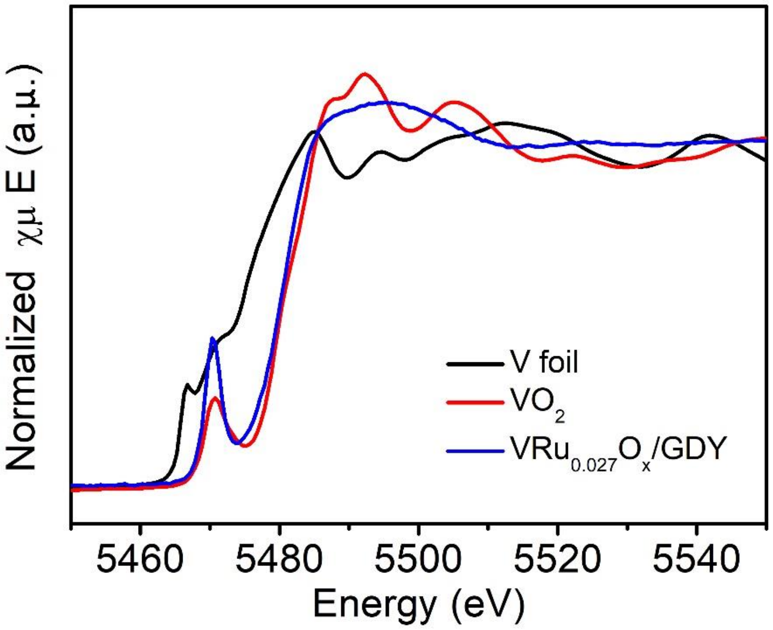

Figure S17. The XANES spectra V K-edge EXAFS spectra of V foil, VO_2 and $VRu_{0.027}O_x$/GDY.

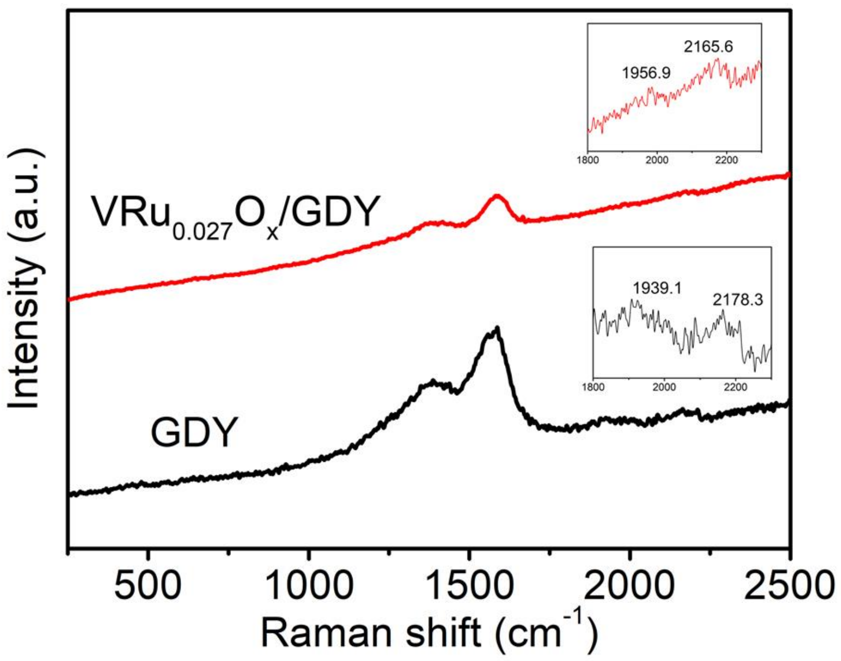

Figure S18. Raman spectroscopy of GDY and $VRu_{0.027}O_x$/GDY.

In the Raman spectra of GDY, the peaks at 1375.5 and 1569.7 cm^{-1} corresponding to D and G bands, and the peaks at 1939.1 and 2178.3 cm^{-1} due to vibrations of the conjugated diyne links were observed. For $VRu_{0.027}O_x$/GDY, the peaks at 1382.5 (D band), 1580.5 cm^{-1} (G band), 1956.9 and 2165.6 cm^{-1} (vibration of the conjugated diyne links) corresponding to the GDY species. From the Raman spectrum, we could not observe the peak of $VRu_{0.027}O_x$, which might be due to the small size of $VRu_{0.027}O_x$ on GDY surface.

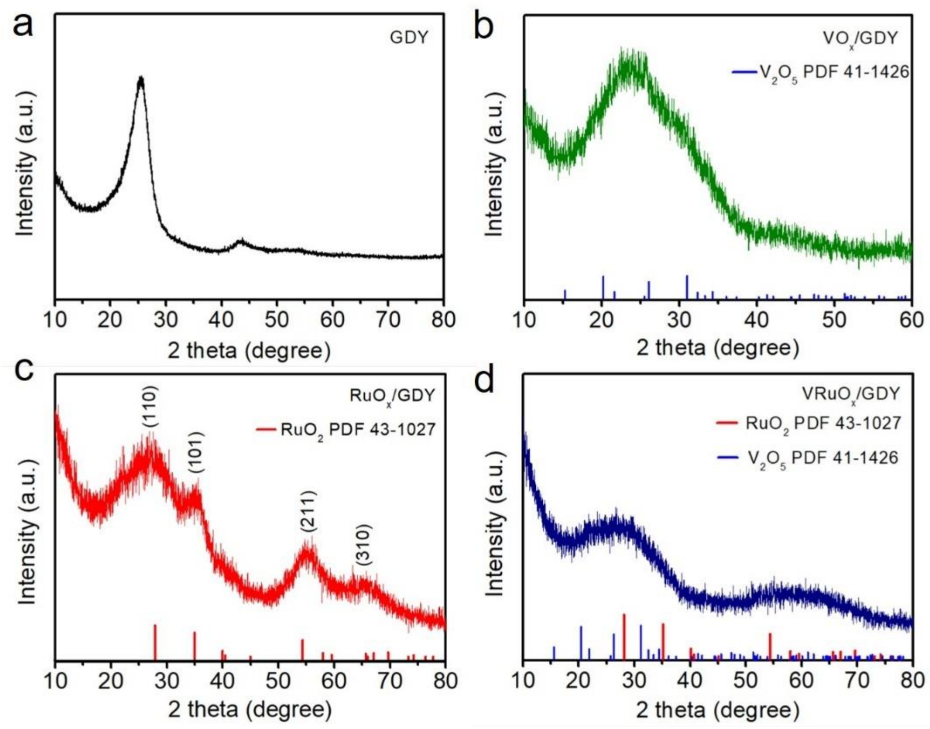

Figure S19. XRD spectra of a) GDY, b) VO_x/GDY, c) RuO_x/GDY and d) $VRu_{0.027}O_x$/GDY.

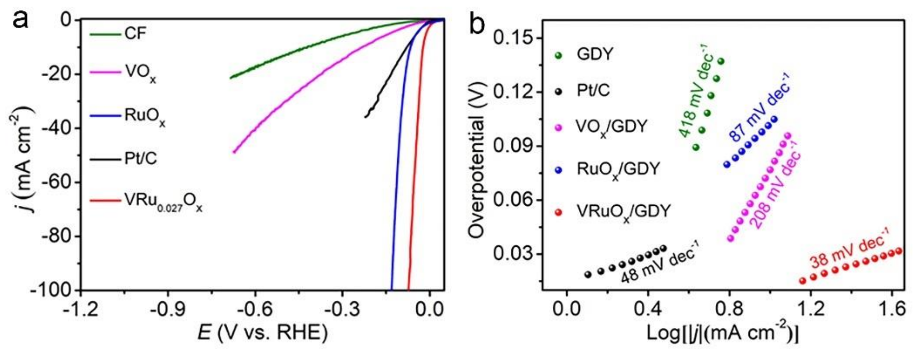

Figure S20. a) Polarization curves and b) corresponding Tafel slopes calculated of the catalysts for HER in 1.0 M KOH.

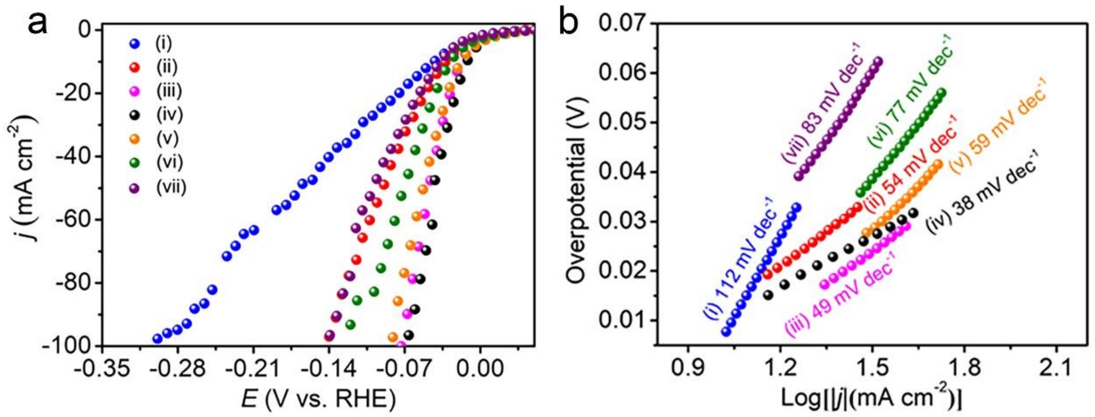

Figure S21. a) Polarization curves and b) Tafel curves of the catalysts (i) $VRu_{0.012}O_x$/GDY, (ii) $VRu_{0.017}O_x$/GDY, (iii) $VRu_{0.022}O_x$/GDY, (iv) $VRu_{0.027}O_x$/GDY, (v) $VRu_{0.032}O_x$/GDY, (vi) $VRu_{0.037}O_x$/GDY and (vii) $VRu_{0.042}O_x$/GDY for HER in 1.0 M KOH.

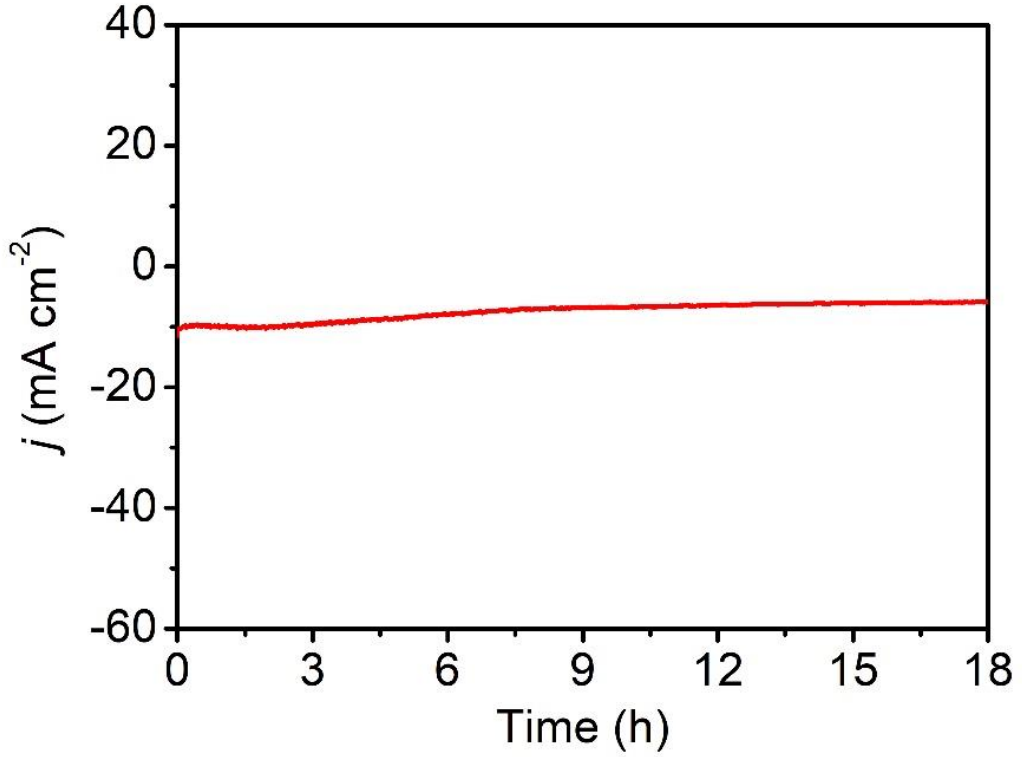

Figure S22. Time-dependent current density curve of $VRu_{0.027}O_x$/GDY at the potential of -0.022V (vs. RHE) in 1.0 M KOH.

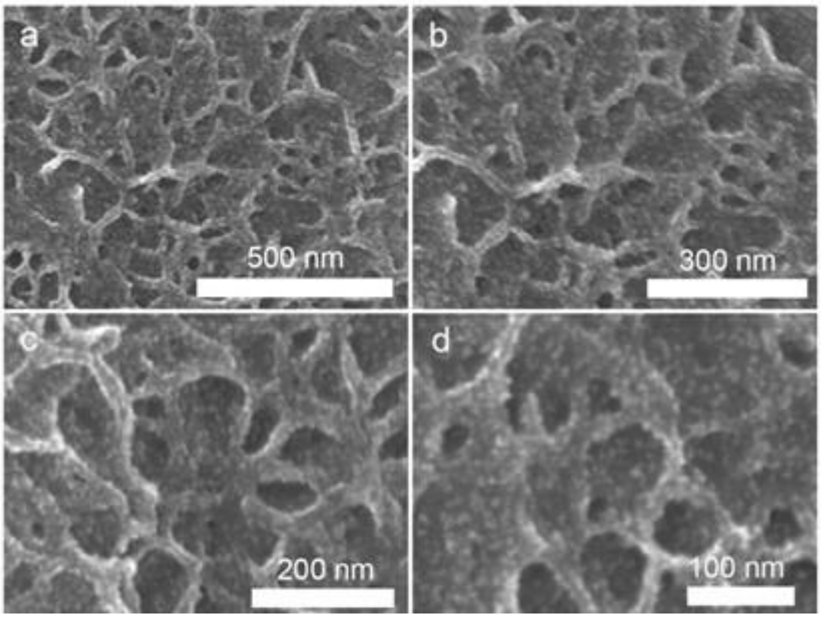

Figure S23. SEM images of $VRu_{0.027}O_x$/GDY after continuous cycling test in 1.0 M KOH condition.

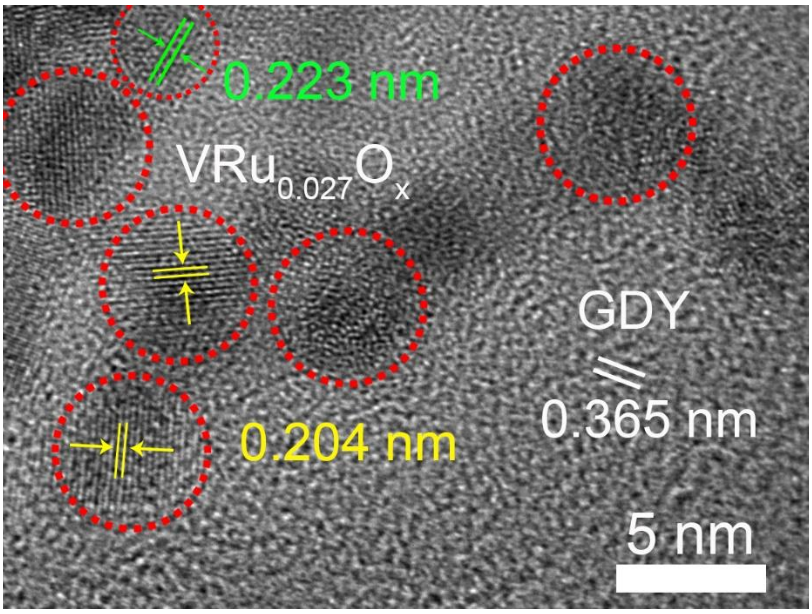

Figure S24. TEM images of $VRu_{0.027}O_x$/GDY after continuous cycling test in 1.0 M KOH condition.

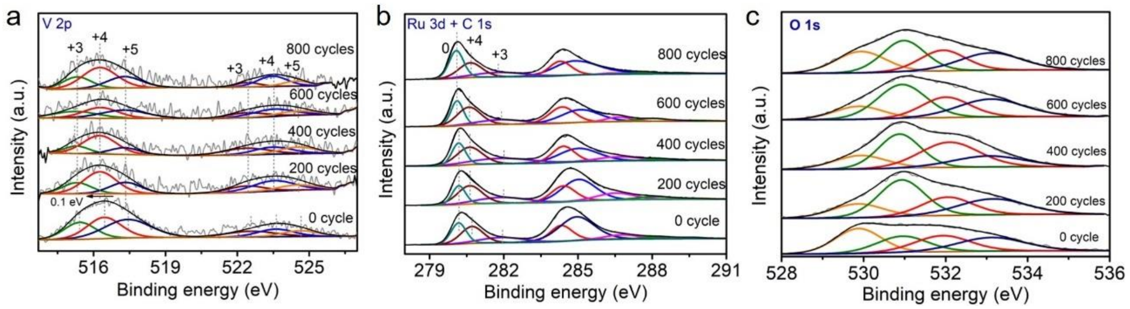

Figure S25. a) V 2p, b) Ru 3d + C 1s and c) O 1s of $VRu_{0.027}O_x$/GDY after cycling tests in 1.0 M KOH.

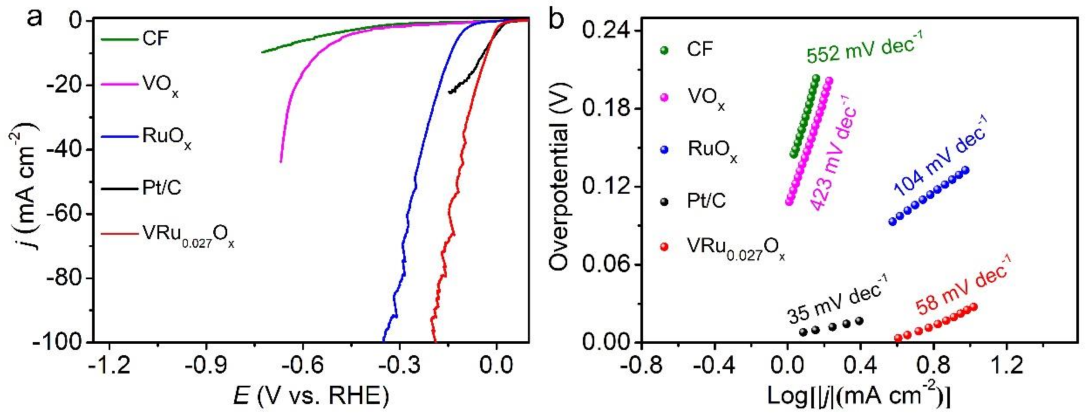

Figure S26. a) Polarization curves and b) corresponding Tafel slopes calculated of the catalysts for HER in 1.0 M PBS.

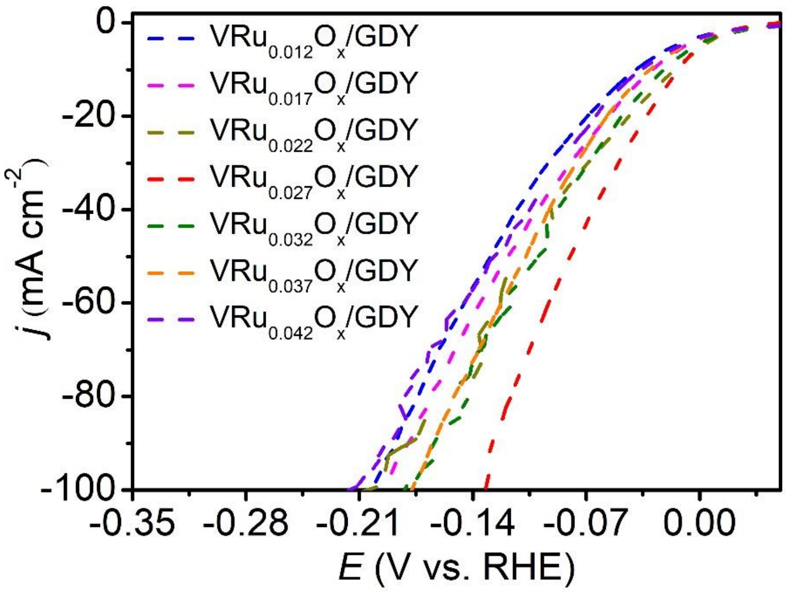

Figure S27. Polarization curves of the catalysts with different Ru/V molar ratios for HER in 1.0 M PBS.

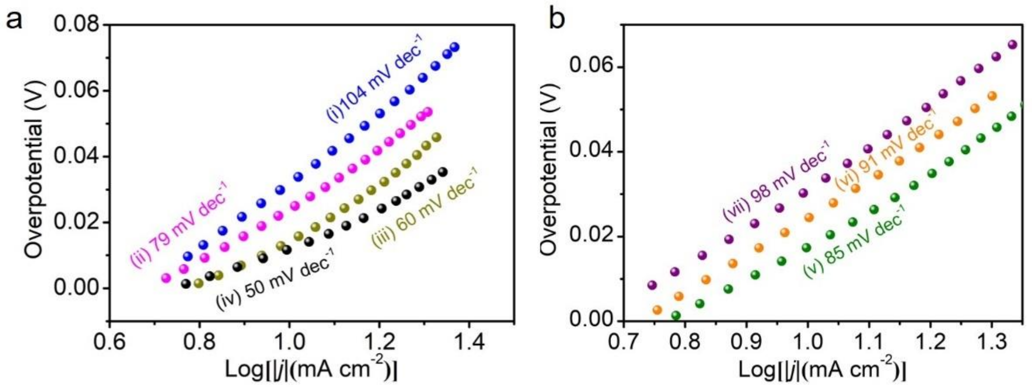

Figure S28. Tafel curves of the catalysts (i) $VRu_{0.012}O_x$/GDY, (ii) $VRu_{0.017}O_x$/GDY, (iii) $VRu_{0.022}O_x$/GDY, (iv) $VRu_{0.027}O_x$/GDY, (v) $VRu_{0.032}O_x$/GDY, (vi) $VRu_{0.037}O_x$/GDY and (vii) $VRu_{0.042}O_x$/GDY for HER in 1.0 M PBS.

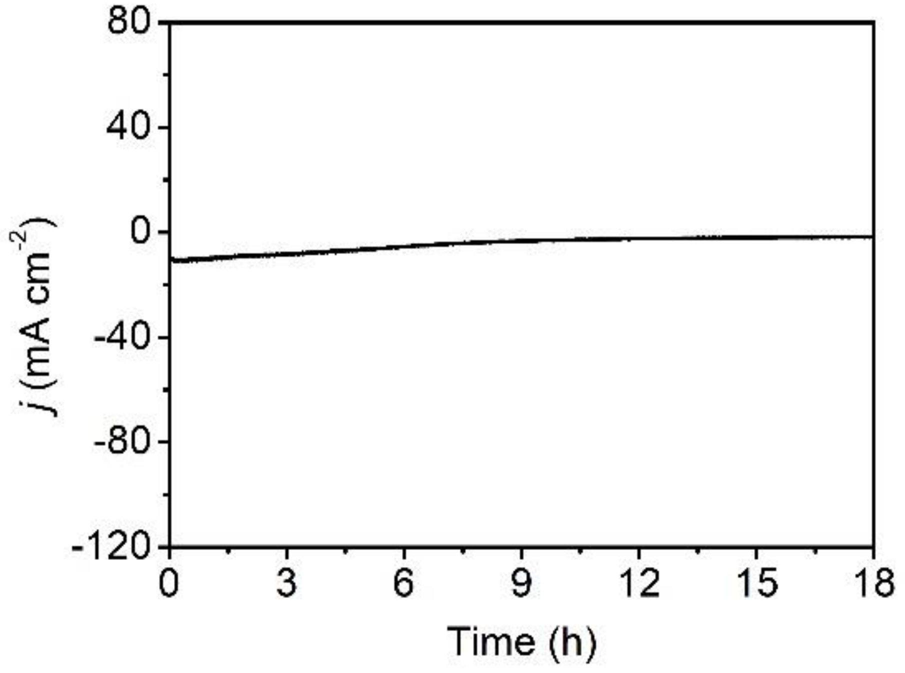

Figure S29. Time-dependent current density curve of $VRu_{0.027}O_x$/GDY in 1.0 M PBS.

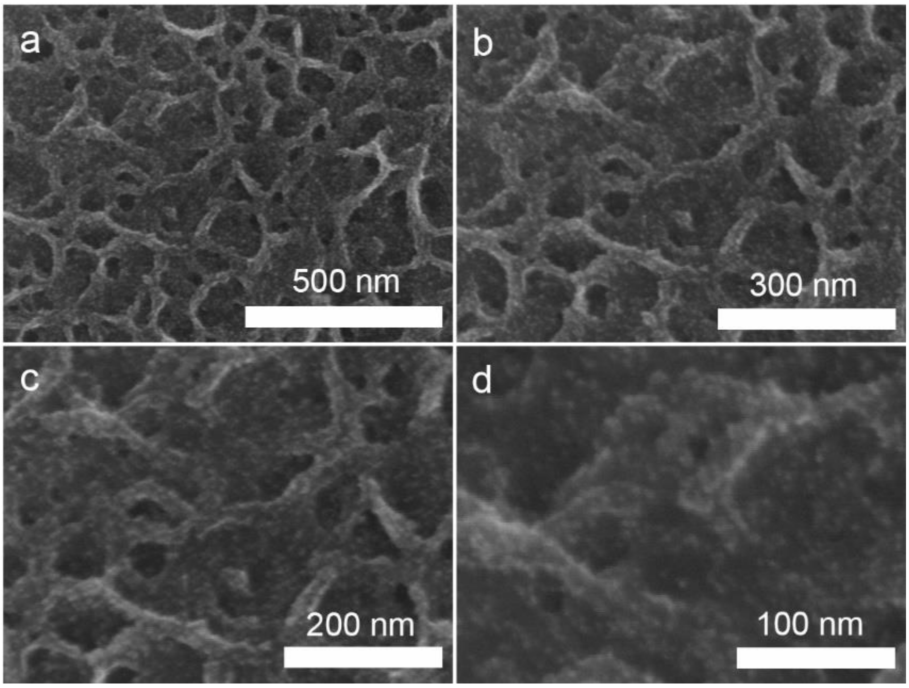

Figure S30. SEM images of $VRu_{0.027}O_x$/GDY after long term stability test in 1.0 M PBS condition.

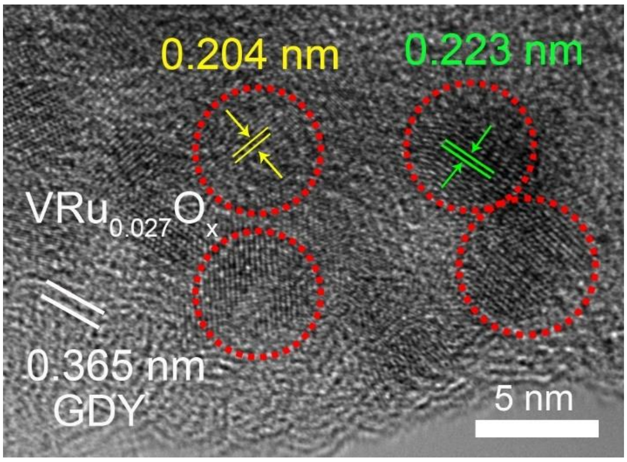

Figure S31. HRTEM image of $VRu_{0.027}O_x$/GDY after cycling tests in 1.0 M PBS.

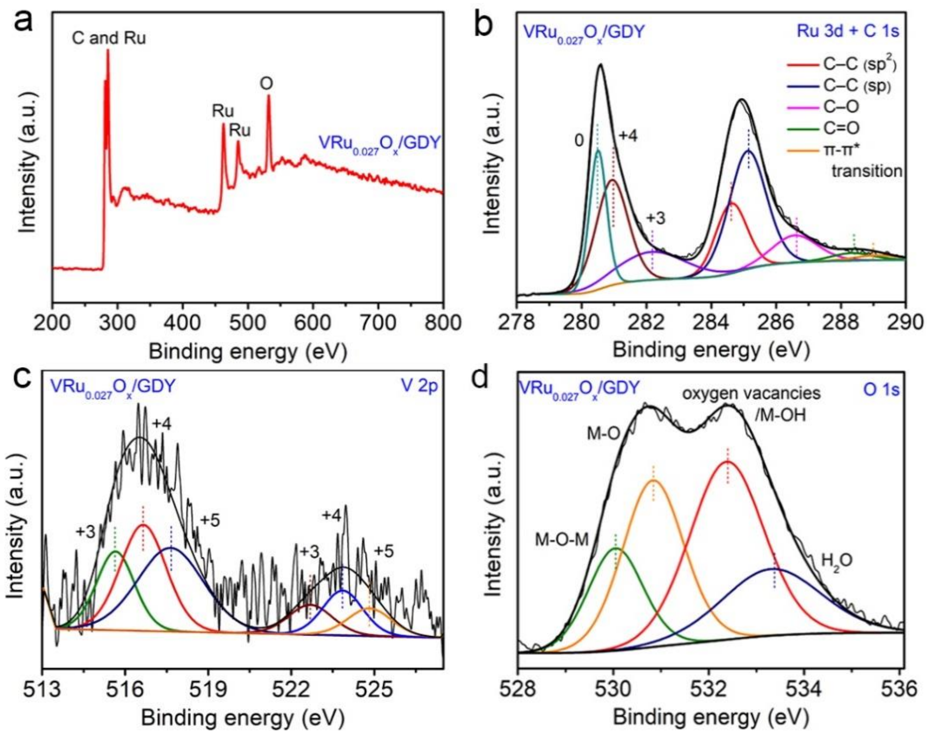

Figure S32. High resolution a) XPS survey spectrum, b) C 1s and Ru 3d, c) V 2p and d) O 1s spectrum of $VRu_{0.027}O_x$/GDY after cycling tests in 1.0 M PBS.

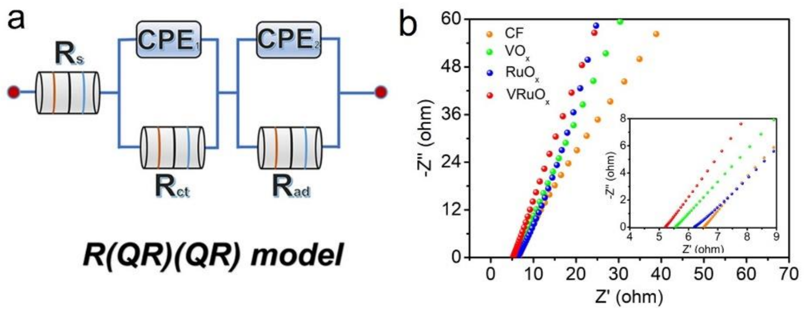

Figure S33. a) R(QR)(QR) equivalent circuit model b) Nyquist plots of the catalysts of VO_x, RuO_x, $VRu_{0.027}O_x$ and CF, respectively.

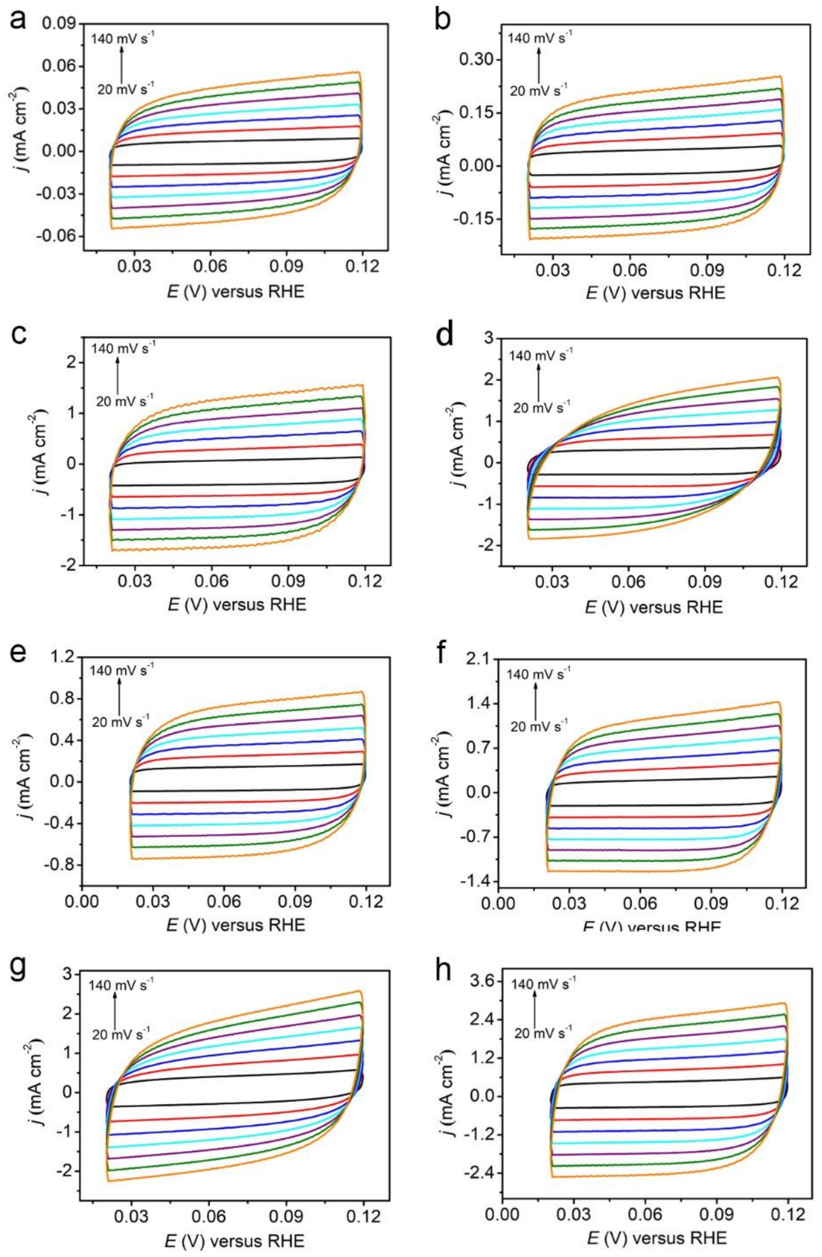

Figure S34. CV measurements of a) CF, b) GDY, c) RuO_x, d) RuO_x/GDY, e) VO_x, f) VO_x/GDY, g) $VRu_{0.027}O_x$ and h) $VRu_{0.027}O_x$/GDY at different scan rate (20, 40, 60, 80, 100, 120 and 140 mV s^{-1}) for C_{dl} determination.

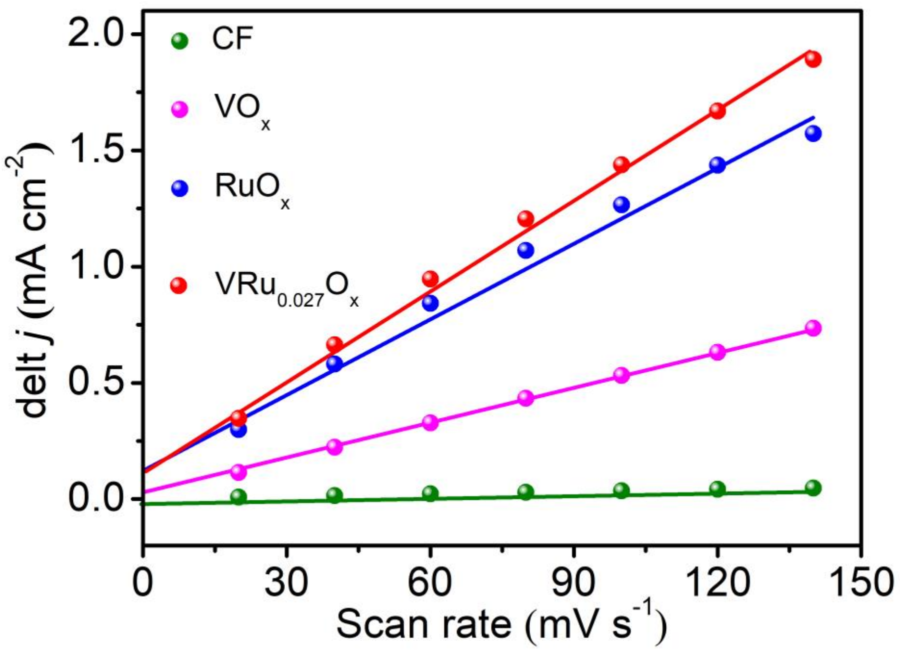

Figure S35. Extraction of the doublelayer capacitance (C_{dl}) for catalysts CF, VO_x, RuO_x and $VRu_{0.027}O_x$ in 1.0 M KOH.

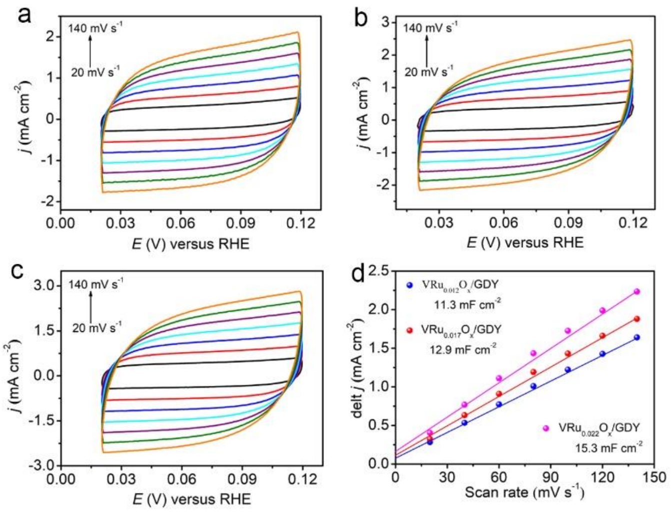

Figure S36. CV measurements of a) $VRu_{0.012}O_x$/GDY, b) $VRu_{0.017}O_x$/GDY and c) $VRu_{0.022}O_x$/GDY at different scan rate (20, 40, 60, 80, 100, 120 and 140 mV s^{-1}) for C_{dl} determination. d) Extraction of the doublelayer capacitance (C_{dl}) for catalysts $VRu_{0.012}O_x$/GDY, $VRu_{0.017}O_x$/GDY and $VRu_{0.022}O_x$/GDY in 1.0 M KOH.

As shown in Figure S36, the C_{dl} value of $VRu_{0.012}O_x$/GDY, $VRu_{0.017}O_x$/GDY and $VRu_{0.022}O_x$/GDY was 11.3, 12.9 and 15.3 mF cm^{-2}. For calculating the ESCA, we use specific capacitances (C_s) of 0.04 mF cm^{-2} according to previous report. The ESCA for $VRu_{0.012}O_x$/GDY, $VRu_{0.017}O_x$/GDY and $VRu_{0.022}O_x$/GDY were determined to be 335.0, 322.5 and 382.5 cm^{-2}, respectively.

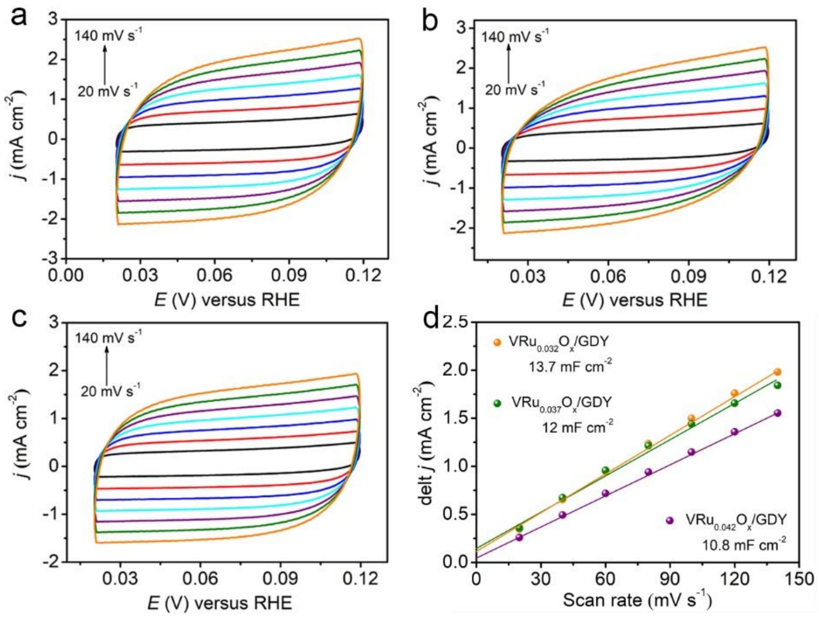

Figure S37. CV measurements of a) $VRu_{0.032}O_x$/GDY b) $VRu_{0.037}O_x$/GDY and c) $VRu_{0.042}O_x$/GDY at different scan rate (20, 40, 60, 80, 100, 120 and 140 mV s^{-1}) for C_{dl} determination. d) Extraction of the doublelayer capacitance (C_{dl}) for catalysts $VRu_{0.032}O_x$/GDY, $VRu_{0.037}O_x$/GDY and $VRu_{0.042}O_x$/GDY in 1.0 M KOH.

As shown in Figure S37, the C_{dl} value of $VRu_{0.032}O_x$/GDY, $VRu_{0.037}O_x$/GDY and $VRu_{0.042}O_x$/GDY was 13.7, 12.0 and 10.8 mF cm^{-2}. For calculating the ESCA, we use specific capacitances (C_s) of 0.04 mF cm^{-2} according to previous report. The ESCA value for $VRu_{0.032}O_x$/GDY, $VRu_{0.037}O_x$/GDY and $VRu_{0.042}O_x$/GDY was 342.5, 300.0 and 270.0 cm^{-2}.

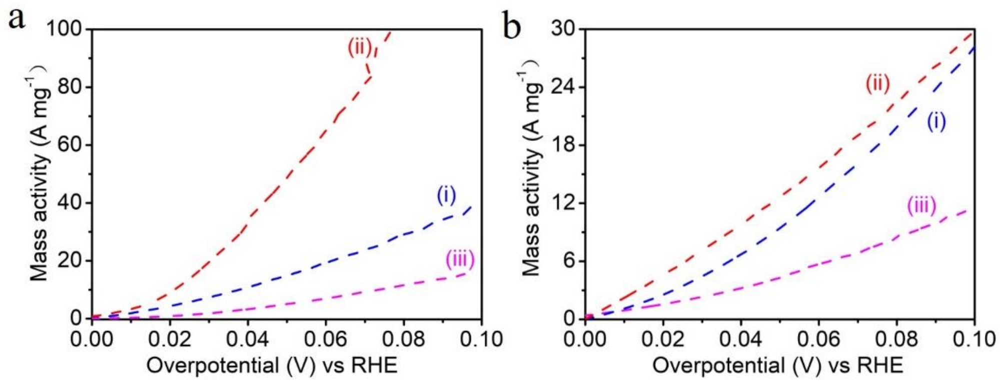

Figure S38. a) Mass activities of (i) $VRu_{0.012}O_x$/GDY, (ii) $VRu_{0.027}O_x$/GDY, and (iii) $VRu_{0.042}O_x$/GDY in 1.0 M KOH. b) Mass activities of (i) $VRu_{0.012}O_x$/GDY, (ii) $VRu_{0.027}O_x$/GDY, and (iii) $VRu_{0.042}O_x$/GDY in 1.0 M PBS.

As shown in Figure S38, $VRu_{0.027}O_x$/GDY possesses a better mass activity toward HER than $VRu_{0.012}O_x$/GDY and $VRu_{0.042}O_x$/GDY in 1.0 M KOH and 1.0 M PBS, respectively. At the verpotential of 0.05 V, the mass activities of $VRu_{0.012}O_x$/GDY, $VRu_{0.027}O_x$/GDY and $VRu_{0.042}O_x$/GDY are 15.2, 49.1 and 5.10 A mg_{metal}^{-1}, respectively, in 1.0 M KOH. At the overpotential of 0.05 V, the mass activities of $VRu_{0.012}O_x$/GDY, $VRu_{0.027}O_x$/GDY and $VRu_{0.042}O_x$/GDY are 10.3, 12.7 and 4.3 A mg_{metal}^{-1}, respectively, in 1.0 M PBS.

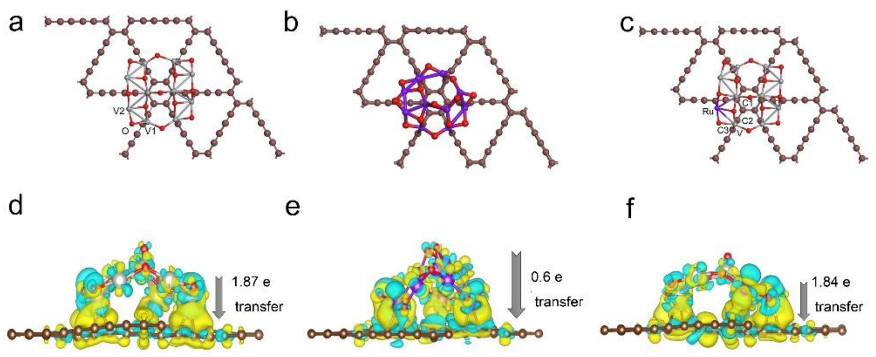

Figure S39. Optimized configurations of graphdiyne (GDY) loaded a) VO_x, b) RuO_x and c) $VRu_{0.027}O_x$. Charge distribution in d) VO_x/GDY, e) RuO_x/GDY, and f) $VRu_{0.027}O_x$/GDY (Brown, silver, purple, and red balls represent C, V, Ru, and O atoms, respectively).

Table S1. The proportion of the fitted subpeaks for $VRu_{0.027}O_x$/GDY and GDY samples.

Samples	sp－C	sp^2－C	C－O	C=O	π-π* transition
$VRu_{0.027}O_x$/GDY	25.6%	51.2%	13.3%	7.7%	2.2%
GDY	26.5%	56.1%	11.2%	6%	0

Table S2. The Ru/V molar ratios and corresponding Ru and V atomic contents based on the XPS spectra of $VRuO_x$/GDY.

**Samples**	**Ru/V molar ratios**	**Ru atomic**	**VV atomic**
**$VRu_{0.012}O_x$/GDY**	0.012	1.58	0.28
**$VRu_{0.017}O_x$/GDY**	0.017	27.41	3.16
**$VRu_{0.022}O_x$/GDY**	0.022	33.15	3
**$VRu_{0.027}O_x$/GDY**	0.027	11.71	1.45
**$VRu_{0.032}O_x$/GDY**	0.032	15.43	1.6
**$VRu_{0.037}O_x$/GDY**	0.037	33.59	2.89
**$VRu_{0.042}O_x$/GDY**	0.042	44.6	4.33

Table S3. The percentage of lattice oxygen (O1), metal-oxygen (O2), surface-adsorbed oxygen (O3) and adsorbed molecular water (O4) based on the XPS spectra of catalysts VO_x/GDY, RuO_x/GDY and $VRu_{0.027}O_x$/GDY.

Samples	O1	O2	O3	O4
**VO_x/GDY**	0	19.0%	52.8%	28.2%
**RuO_x/GDY**	0	25%	33.6%	41.4%
**$VRu_{0.027}O_x$/GDY**	30.2%	21.7%	23.8%	24.3%

Table S4. The overpotentials (η) for all catalyst in 1.0 M KOH solution to achieve the current density of 10 mA cm^{-2}, 50 mA cm^{-2} and 100 mA cm^{-2}, respectively.

**Samples**	η (mV) @10 mA cm^{-2}	η (mV) @50 mA cm^{-2}	η (mV) @100 mA cm^{-2}
$VRu_{0.012}O_x$/GDY	43	167	279
$VRu_{0.017}O_x$/GDY	32	90	144
$VRu_{0.022}O_x$/GDY	18	48	73
$VRu_{0.027}O_x$/GDY	13	43	69
$VRu_{0.032}O_x$/GDY	18	53	82
$VRu_{0.037}O_x$/GDY	28	71	108
$VRu_{0.042}O_x$/GDY	37	99	151
CF	420	–	–
GDY	340	–	–
RuO_x	66	108	132
RuO_x/GDY	46	99	140
VO_x	238	–	–
VO_x/GDY	112	289	432
$VRu_{0.027}O_x$	26	50	74

Table S5. Comparison of overpotential (η) at current density of 10 mA cm^{-2} and Tafel slopes of $VRu_{0.027}O_x$/GDY with recently reported catalysts in 1.0 M KOH aqueous solution.

Catalyst	η at j = 10 mA cm^{-2} (mV)	Tafel slope (mV dec^{-1})	η at j = 100 mA cm^{-2} (mV)	Mass loading (ug cm^{-2})	Ref.
$VRu_{0.027}O_x$GDY	**13**	**38**	**69**	**2.32**	**This work**
Sr_2RuO_4	61	51	N/A	N/A	*Nat. Commun.* **2019**, *10*, 149.
Ru NCS/BNG	14	28.9	N/A	N/A	*Nano Energy* **2020***, 68,* 104301
Ru-Mo_2N	16	35	N/A	1000.0	*Nano Energy* **2020***, 75,* 104981
Ru@MWCNT	17	27	N/A	N/A	*Nat. Commun.* **2020***,11,1278.*
Ru-ZIF-900	51.6	78.4	N/A	N/A	*J. Mater. Chem. A* **2020**, *8*, 3203.
Ni_5P_4-Ru/CC	54	52	N/A	N/A	*Adv. Mater.* **2020**, *32*, 1906972
Cu-Ru alloy	15	30	N/A	N/A	*ACS Energy Lett.* **2020**, *5*, 192
RuNi/CQDs	13	40	N/A	N/A	*Angew. Chem. Int. Ed.* **2020**, *59*, 1 718
(Ru-Co)O_x	44.1	23.5	89.1	N/A	Angew. Chem. Int. Ed. **2020**, *59*, 17219
Ru SAs/N-Mo_2C	43	38.67	N/A	N/A	*Applied Catalysis B: Environmental* **2020**, *277*, 119236
RuS_x/s-GO	58	56	N/A	N/A	*Small* **2019**, *15*, 1904043
MoS_2	332	119	N/A	N/A	*Nat. Commun.* **2020**, *11,* 1116
Ni/Fe NP	112	212	N/A	N/A	*Nat. Commun.* **2019**, *10*, 5599
NiV-LDH/NF	209	114	N/A	N/A	*Nat. Commun.* **2019**, *10*, 3899.
Co/β-Mo_2C@N-CNTs	170	92	N/A	N/A	*Angew. Chem. Int. Ed.* **2019**, *58*, 4923.
Fe-Ni@NC-CNTs	202	113.7	N/A	N/A	*Angew. Chem. Int. Ed.* **2018**, *57*, 8921.
RuP_2@NPC	52	69	N/A	N/A	*Angew. Chem. Int. Ed.* **2017**, *56*, 11559.
$NiCo_2S_4$	80 mV	58.5	N/A	N/A	*Adv. Funct. Mater.* **2019**, 1807031.

W_2N/WC	148.5	47.4	N/A	N/A	*Adv. Mater*. **2020**, *32*, 1905679.
CoFeZr	104	119.3	N/A	N/A	*Adv. Mater.* **2019**, *31*, 1901439.
EBP@NG(1:8)	210	109	N/A	N/A	*J. Am. Chem. Soc.* **2019**, *141*, 4972.
CoMoNiS-NF-31	113	85	N/A	N/A	*J. Am. Chem. Soc.* **2019**, *141*, 10417.
Ni-Co-O	260	127	N/A	N/A	*J. Am. Chem. Soc.* **2018**, *140*, 5241.
VOOH	164	104	N/A	N/A	*Angew. Chem. Int. Ed*. **2017**, *56*, 573.

Table S6. The overpotentials of all catalyst in 1.0 M PBS solution to achieve the current density of 10 mA cm^{-2}, 50 mA cm^{-2} and 100 mA cm^{-2}, respectively.

**Samples**	η (mV) @10 mA cm^{-2}	η (mV) @50 mA cm^{-2}	η (mV) @100 mA cm^{-2}
$VRu_{0.012}O_x$/GDY	40	129	202
$VRu_{0.017}O_x$/GDY	30	118	194
$VRu_{0.022}O_x$/GDY	16	109	205
$VRu_{0.027}O_x$/GDY	12	80	132
$VRu_{0.032}O_x$/GDY	22	100	181
$VRu_{0.037}O_x$/GDY	31	107	177
$VRu_{0.042}O_x$/GDY	37	127	213
CF	–	–	–
GDY	727	–	–
RuO_x	142	252	349
RuO_x/GDY	137	266	470
VO_x	597	–	–
VO_x/GDY	552	–	–
$VRu_{0.027}O_x$	29	122	192

Table S7. Comparison of overpotential (η) at current density of 10 mA cm^{-2} and Tafel slopes of $VRu_{0.027}O_x$/GDY with recently reported catalysts in 1.0 M PBS aqueous solution.

Catalysts	η at j = 10 mA cm^{-2} (mV)	Tafel slope (mV dec^{-1})	Ref.
**$VRu_{0.027}O_x$/GDY**	**12**	**50**	**This work**
CoMoNiS-NF-31	117	56	*J. Am. Chem. Soc.* **2019**, *141*, 10417.
RuP_2@NPC	57	87	*Angew. Chem. Int. Ed.* **2017**, *56*, 11559.
Ru@SC-CDs	66		*Nano Energy* **2019**, *65*, 104023.
V-CPNA/CC	98	83.7	*ACS Appl. Energy Mater.* **2020**, *3*, 1027.
$(Fe_{0.048}Ni_{0.952})_2P$	90	82.7	*Nano Energy* **2019**, *56,* 813.
Co_xNi_yN	152.8	90.32	*ACS Appl. Mater. Interfaces* **2019**, *11*, 8018.
N-Co_2P/CC	42	68	*ACS Catal.* **2019**, *9*, 3744.
Mo_2C-GNR-2	116	64	*ACS Sustainable Chem. Eng.* **2016**, *4*, 6313.
Ni/β-Mo_2C	149	66	*Chem. Commun.* **2018**, *54,* 9901.
CoP-400	161	81	*Small* **2018**, *14*, 1802824
NiSe@NC-600	300	66.2	*Nanoscale* **2018**, *10*, 22758.
Mo-Ni_2P/NF	84	85	*Nanoscale* **2017**, *9*, 16674.

Table S8. The values of resolution resistance (R_s) and charge transfer resistances (R_{ct}) of catalysts in 1.0 M KOH at 298 K using R(QR)(QR) equivalent circuit model.

**Catalysts**	**CF**	**GDY**	**RuO_x/ GDY**	**VO_x/ GDY**	**$VRu_{0.027}O_x$ /GDY**
R_s	6.597	6.46	5.518	5.27	4.95
R_{ct}	3929	1821	328	156	50.3

Table S9. The electrochemical active surface area (ECSA) and specific current densities (j_s) of samples.

Catalysts	ESCA (cm^2)	R_f	j_s (mA cm^{-2} at η = -50 mV for HER (1.0 M KOH)
**$VRu_{0.012}O_x$/GDY**	283	35.3	0.0046
**$VRu_{0.017}O_x$/GDY**	323	16.2	0.0241
**$VRu_{0.022}O_x$/GDY**	383	23.9	0.0316
**$VRu_{0.027}O_x$/GDY**	430	21.8	0.0321
**$VRu_{0.032}O_x$/GDY**	343	17.2	0.0319
**$VRu_{0.037}O_x$/GDY**	300	18.8	0.0172
**$VRu_{0.042}O_x$/GDY**	270	14.21	0.0123

The specific current densities (j_s) for our catalysts can be calculated by dividing the current density per geometric area (j_g) at a given overpotential by the determined roughness factor (R_f), which was calculated by dividing electrochemically active surface area (ECSA) by geometric area of the electrode.

R_f was calculated by taking the estimated ECSA and dividing it by the geometric area of the electrode.

R_f = ESCA / Geometricarea
